# PRedicting Outcomes For Crohn’s dIsease using a moLecular biomarkEr (PROFILE): protocol for a multicentre, randomised, biomarker-stratified trial

**DOI:** 10.1136/bmjopen-2018-026767

**Published:** 2018-12-05

**Authors:** Miles Parkes, Nurulamin M Noor, Francis Dowling, Harvey Leung, Simon Bond, Lynne Whitehead, Sara Upponi, Paul Kinnon, Andrew P Sandham, Paul A Lyons, Eoin F McKinney, Kenneth G C Smith, James C Lee

**Affiliations:** 1 Department of Medicine, University of Cambridge School of Clinical Medicine, Addenbrooke’s Hospital, Cambridge, UK; 2 Cambridge Clinical Trials Unit, Cambridge University Hospitals NHS Foundation Trust, Cambridge, UK; 3 Medical Research Council Biostatistics Unit, University of Cambridge, Cambridge, UK; 4 Clinical Trials Pharmacy, Addenbrooke’s Hospital, Cambridge, UK; 5 Department of Radiology, Addenbrooke’s Hospital, Cambridge, UK; 6 PredictImmune Ltd, Babraham Research Campus, Cambridge, UK

**Keywords:** genetics, inflammatory bowel disease, immunology, gastroenterology, clinical trials

## Abstract

**Background:**

The course of Crohn’s disease (CD) varies substantially between individuals, but reliable prognostic markers do not exist. This hinders disease management because patients with aggressive disease are undertreated by conventional ‘step-up’ therapy (in which treatment is gradually escalated in response to refractory or relapsing disease) while those with more indolent disease would be exposed to unnecessary treatment-related toxicity if a more aggressive ‘top-down’ approach was indiscriminately used. The Predicting outcomes for Crohn’s disease using a molecular biomarker trial will assess whether a prognostic transcriptional biomarker, that we have developed and validated, can improve clinical outcomes by facilitating personalised therapy in CD. This represents the first the biomarker-stratified trial in inflammatory bowel disease.

**Methods and analysis:**

This biomarker-stratified trial will compare the relative efficacy of ‘top-down’ and ‘accelerated step-up’ therapy between biomarker-defined subgroups of patients with newly diagnosed CD. 400 participants from ~50 UK centres will be recruited. Subjects within each biomarker subgroup (IBD^hi^ or IBD^lo^) will be randomised (1:1) to receive one of the treatment strategies until trial completion (48 weeks). The primary outcome is the incidence of sustained surgery and steroid-free remission from the completion of induction treatment through to week 48. Secondary outcomes include mucosal healing, quality-of-life assessments and surrogate measures of disease burden including number of flares, cumulative steroid exposure, number of hospital admissions and number of Crohn’s-related surgeries (assessed hierarchically). Analyses will compare the relative benefit of the treatment strategies in each biomarker-defined subgroup, powered as an interaction analysis, to determine whether the biomarker can accurately match patients to the most appropriate therapy.

**Ethics and dissemination:**

Ethical approval has been obtained and recruitment is under way at sites around the UK. Following trial completion and data analysis, the results of the trial will be submitted for publication in peer-reviewed journals and presented at international conferences.

**Trial registration number:**

ISRCTN11808228; Pre-results.

**Strengths and limitations of this study**

- The first biomarker-stratified trial in inflammatory bowel disease, comparing the relative benefit of ‘top-down’ over ‘accelerated step-up’ therapy in biomarker-defined subgroups of patients with newly diagnosed Crohn’s disease.
- The largest interventional trial ever conducted in adult patients with newly diagnosed Crohn’s disease, incorporating 400 patients across approximately 50 sites.
- Findings have the potential to demonstrate that personalised therapy can be effectively delivered to patients with Crohn’s disease at the time of diagnosis using a blood-based prognostic biomarker.
- Study limited to the UK.
- Top-down therapy limited to treatment with infliximab and an immunomodulator (which may be superseded by other treatments in the future).

## Introduction

Crohn’s disease (CD) is a relapsing-remitting form of inflammatory bowel disease (IBD) that can affect any part of the intestine, most commonly the ileum and/or colon. It is a common condition, affecting ~1 in 400–500 people in Northwestern Europe and North America, with a steadily rising global incidence.^1 2^

Like many other immune-mediated diseases, the course of CD varies substantially between affected individuals, but no reliable prognostic markers currently exist. The most common treatment strategy in CD is therefore based on a reactive, stepwise escalation in therapy that occurs in response to recurrent flares or persistently active disease. This approach (termed ‘step-up’) should not overtreat patients but will inevitably expose some individuals to cumulative intestinal damage and disease-related complications while therapies that are insufficiently potent for them are trialled.

In 2008, it was shown that early use of anti-tumour necrosis factor α (TNFα) monoclonal antibodies (anti-TNFα therapy) was superior to conventional step-up management.^3^ Further support for early anti-TNFα use came from registration trials, which demonstrated greater efficacy of anti-TNFα therapy when it was used earlier in the disease course^4 5^; and the SONIC trial, which showed that combining anti-TNFα (infliximab) with azathioprine (termed combination or ‘top-down’ therapy) achieved results superior to either alone.^6^ However, it is widely recognised that the indiscriminate use of combination therapy in all patients would expose those patients destined for mild disease to the risks and side-effects of treatment that their disease did not require, and would also be economically unfeasible.

In an attempt to reconcile these issues, subsequent trials have sought to identify approaches that could still deliver relatively early, aggressive therapy but also be economically feasible. The Randomised Evaluation of an Algorithm for Crohn’s Treatment (REACT) trial, for example, investigated whether accelerating more quickly up the treatment ladder (‘accelerated step-up’) would lead to better outcomes.^7^ Similarly, the AZathioprine for Treatment or Early Crohn’s disease in adults (AZTEC) and Résultat de l’Adjonction Précoce d’ImmunoDépresseurs (RAPID) trials investigated whether initiating azathioprine, a less potent but cheaper immunomodulator, in all patients at diagnosis would improve outcomes.^8 9^ However, none of these studies have demonstrated improved efficacy over standard care, leading many to conclude that a ‘precision’ (or ‘personalised’) approach would be required in which the most potent treatments are targeted to those who need them. Unfortunately, despite investigation into the prognostic utility of clinical, genetic and serological markers, there remain no well-validated prognostic tools for CD that can reliably predict the disease course from diagnosis. Indeed, a recent priority setting partnership group, tasked with identifying major areas of unmet need in IBD research, designated the need to develop markers to guide treatment for individual patients as the most important unmet need in IBD.^10^ Consistent with this, a survey of 52 US and 50 UK gastroenterologists (commissioned through Apex Healthcare Consulting) showed that nearly all gastroenterologists recognised a need for an assay that could predict the clinical outcome and probability of relapse in CD (UK 98%, US 94%; table 1). Moreover, if the results of such a biomarker enabled gastroenterologists to amend their treatment approach, all of the respondents would use the test in their practice (table 1).

**Table 1:** Summary results of an independent 2015 survey of practising gastroenterologists performed by Apex Healthcare Consulting

	UK (n=50)	USA (n=52)
‘CD patients are at moderate-to-high risk of relapse throughout their lives’	Agree—80% (40)	Agree—79% (41)
‘There is a need for an assay that would predict clinical outcome and probability of relapse in CD’	Agree—98% (49)	Agree—94% (49)
Would you use a test to predict clinical outcome and probability of relapse even if you could not change your treatment approach?	Yes—58% (29)	Yes—54% (28)
Would you use a test to predict clinical outcome and probability of relapse if it enabled you to alter your treatment approach?	Yes—100% (50)	Yes—100% (52)
How many days following a test to predict clinical outcome and probability of relapse would you require the results for this to be useful?	10 days (mean)	9 days (mean)

Gastroenterologists: clinically active attending physicians (USA) or consultants (UK) with 5–30 years specialty experience, including IBD caseload. Survey funded by Wellcome Trust (Interim Translational Award 099450/Z/12/Z).

CD, Crohn’s disease; IBD, inflammatory bowel disease.

Our group has previously identified a gene expression signature in peripheral blood CD8 +T cells from patients with active, untreated IBD (and other autoimmune diseases) that is related to T cell exhaustion and which correlates with subsequent prognosis.^11–13^ Patients in the IBD1 subgroup, defined by this signature, had a much more aggressive disease than those in the IBD2 subgroup, with earlier recurrence of disease and more flares over time.^11^ To help translate this to routine clinical practice, we have since developed a whole blood qPCR assay that can identify patient subgroups which are analogous to those identified by the CD8 signature, but which does not require cell separation (manuscript in preparation). This assay has been independently validated in prospectively-collected cohorts of UC and CD patients from four centres around the UK.^14^ We now propose to conduct a biomarker-stratified trial to determine whether this biomarker can facilitate the delivery of personalised medicine in CD and improve outcomes.

This manuscript summarises the approved PRedicting Outcomes For Crohn’s dIsease using a moLecular biomarkEr (PROFILE) trial protocol that is in use at the time of publication (V.3.0, 30 April 2018). The full version of the protocol is available at: http://www.crohnsprofiletrial.com/index.php/investigators/downloads/.

The PROFILE trial participant information sheet (PIS) that is in use at the time of publication (V.3.1, 25 June 2018) is available at: http://www.crohnsprofiletrial.com/index.php/participants/downloads/.

Any future amendments to this protocol or PIS will require agreement with the sponsors and amendments will only be initiated following approval by a Research Ethics Committee.

### Aims and objectives

The PROFILE trial will test whether stratification using a whole blood gene expression biomarker can facilitate personalised therapy in CD and improve clinical outcomes. The hypothesis is that the biomarker will identify individuals destined to run an aggressive, relapsing course, and that in these individuals a greater benefit of early top-down therapy will be observed. Similarly, we hypothesise that the biomarker will reliably identify those patients destined to experience more indolent disease, who can be effectively managed using conventional accelerated step-up approaches without the risks and side-effects of unnecessary immunosuppression.

In addition, the trial will seek to advance scientific understanding of CD through the collection of a range of biological samples for future exploratory translational and scientific studies. These will include microbial, metabolomic, proteomic, genetic and transcriptomic samples.

### Methods and analysis

#### Trial design and flow chart

**Figure 1:**
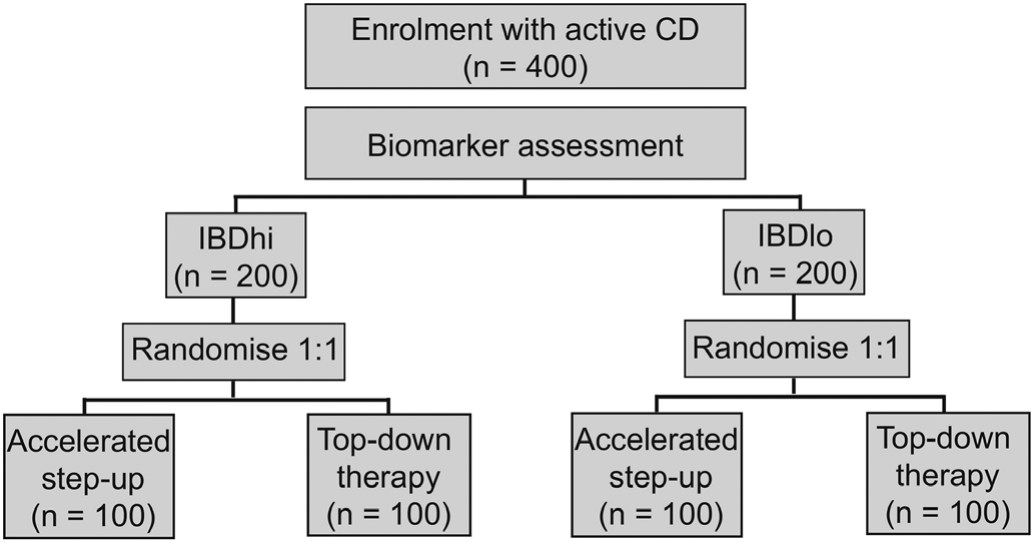
Trial design. Following biomarker stratification, patients will be randomised in a 1:1 fashion to either ‘top-down’ or ‘accelerated step-up’ treatment arms. CD, Crohn’s disease; IBD, inflammatory bowel disease.

The trial is designed as a randomised, biomarker-stratified trial to assess the relative benefit of different treatment approaches in biomarker-defined subgroups. This is an established design for the validation of predictive biomarkers,^15^ and has been used widely in the setting of oncology trials.^16^ Within each biomarker group, patients will be randomised in a 1:1 ratio to receive either top-down or accelerated step-up therapy (figure 1).

#### Trial sites

PROFILE is a multicentre trial based in National Health Service hospitals within the UK. This trial aims to recruit 400 participants with newly diagnosed CD and will be conducted in approximately 50 sites (http://www.crohnsprofiletrial.com/index.php/investigators/).

#### Trial duration

After providing informed consent, participants will be enrolled within the trial for 48 weeks following the baseline visit. There will be a total of six mandatory trial visits, during which data will be collected. These will take place at the same timepoints for all participants and have been timed to coincide with infliximab infusion visits where possible (for those receiving top-down therapy). The end of the trial will be the last participant’s last visit.

#### Eligibility criteria

Patients will be considered eligible for enrolment if they fulfil all of the inclusion criteria and meet none of the exclusion criteria (box 1). The target population are patients with newly diagnosed, active CD who are immunomodulator and anti-TNFα treatment naïve.

#### Patient and public involvement

The development and advancement of personalised medicine in CD represents a major goal for both patients and physicians, and was recently named one of the key research priorities in IBD by a priority setting partnership group, which included both patients and other key stakeholders.^10^

A local panel of patients with CD at Cambridge University Hospitals National Health Service Trust was actively involved in the design of the study and development of study documentation, and feedback was also obtained by a broader panel of non-IBD patients convened by the Cambridge Clinical Trials Unit. Patient support groups (Crohn’s and Colitis UK) were engaged during the conduct of the trial via invitation to investigator meetings, presentation to patient support groups, and publicity of the trial on their website and social media platforms. Both Crohn and Colitis UK and trial participants have also contributed to the content of the trial website (http://www.crohnsprofiletrial.com/index.php/participants), although patients were not directly involved in the recruitment to, or conduct of, the trial.

Following trial completion and reporting, results of the trial will be disseminated in an easy-to-understand format to all trial participants and to Crohn’s and Colitis UK, as well to the general public via press releases and the public engagement team at the University of Cambridge.

Box 1 **Eligibility criteria for the PROFILE trial**

**Inclusion criteria**

Subjects meeting all of the criteria below may be included in the trial:

- CD diagnosed within 3 months using standard endoscopic, histological or radiological criteria*.
- Clinical evidence of active Crohn’s disease (CD) (corresponding to Harvey Bradshaw Index>7).
- Endoscopic evidence of at least moderately active CD (corresponding to Simplified Endoscopic Score in CD>6 or >4 if limited to the terminal ileum).
- C reactive protein>upper limit of normal on local assay or faecal calprotectin>200 µg/g.
- Immunomodulator and anti-TNFα treatment naïve†.
- Aged 16–80 years old.

**Exclusion criteria**

The presence of any of the following would preclude patient inclusion:

- Patients with ulcerative colitis.
- Patients with fistulating perianal CD or active perianal sepsis.
- Patients with obstructive symptoms and evidence of a fixed stricture on radiology or colonoscopy, which suggest that the subject is at high risk of requiring surgery over the following year.
- Patients with contraindications to trial medications.
- Patients who are pregnant or breast feeding at baseline.
- Other serious medical or psychiatric illness currently ongoing, or experienced in the last 3 months, that could compromise the trial.
- Patients unable to comply with protocol requirements (for reasons including alcohol and/or recreational drug abuse).

*Newly diagnosed patchy colonic inflammation, initially diagnosed as indeterminate colitis, would meet inclusion criteria if clinical impression consistent with CD.†Patients need to have discontinued systemic corticosteroids for 1 week or more prior to screening assessments and still have ongoing, active disease.

#### Outcome measures

##### Primary outcome

Incidence of sustained surgery and steroid-free remission from the completion of induction treatment (a standard, 8-week course of oral steroids) through to week 48. (Remission=Harvey Bradshaw Index (HBI)<4. Requirement for a course of systemic glucocorticoids for active CD would result in failure to meet the primary outcome measure.)

##### Secondary outcomes

1. Mucosal healing (assessed using Simplified Endoscopic Score in CD (SES-CD)).
2. Quality-of-life assessment (assessed using IBD Questionnaire).
3. Assessment of cumulative disease burden based on:
  - Number of flares by 1 year.
  - Cumulative glucocorticoid exposure by 1 year.
  - Steroid-free remission by 1 year.
  - Number of hospital admissions and CD surgeries by 1 year.

#### Health economic evaluation

During the course of the trial, there will be a local health economic analysis conducted by the Cambridge Centre for Health Services Research, as well as a national health economic analysis conducted by the National Institute for Health and Clinical Excellence. The findings of these health economic analyses will be disseminated alongside clinical trial findings.

#### Treatment assignment

All patients considered eligible for the trial at the screening visit will have an 8-week reducing course of prednisolone initiated for treatment of their active luminal CD following screening assessments. Each will be assigned a unique participant ID number, for which a biomarker result will be returned. Anonymised data on all participants who are approached will be collated in accordance with Consolidated Standards of Reporting Trials guidelines. Following biomarker assessment, participants in each biomarker subgroup will be randomly assigned (1:1) to either top-down or accelerated step-up therapy, using a computer-generated algorithm (figure 1). This will occur within 14 days of screening (plus or minus 5 days).

As the trial is testing the ability of the biomarker to stratify therapy, rather than the efficacy of the individual medications (which are established treatments for CD), PROFILE has been designated a non-clinical trial of investigational medicine product. All treatments will be open label, but clinicians and participants will be blinded to biomarker subgroup designation.

#### Treatment arms

Following induction treatment with prednisolone, patients will follow the treatment strategy to which they are randomised. These are:

##### Accelerated step-up therapy

- Flare 1 (after induction therapy or if disease reflares during induction therapy): commence azathioprine (2.5 mg/kg) or low-dose 6-mercaptopurine with allopurinol (if mild intolerance to azathioprine) or methotrexate (if severe intolerance to thiopurines or thiopurine methyltransferase (TPMT) null) together with a 12-week reducing course of prednisolone.
- Flare 2: commence infliximab. If suboptimal response, then for infliximab dose escalation as outlined in the full trial protocol.
- Flare 3+ (ie, disease flare after infliximab dose optimisation): 8-week reducing course of prednisolone.

##### Top-down therapy

- Infliximab started 2 weeks after randomisation with azathioprine (2.5 mg/kg) or alternative immunomodulator as described above. If suboptimal response, then for infliximab dose escalation as described in the full trial protocol. The rate of weaning of prednisolone should be accelerated once infliximab is given to 10 mg/week.
- Subsequent disease flares (ie, disease flare after infliximab dose optimisation): 8-week reducing course of prednisolone.

Participants with persistent non-response to infliximab can have early treatment termination and revert to standard care, at the discretion of their local clinical team.

#### Trial procedures and assessments

Newly diagnosed patients with CD will be recruited from a predominantly outpatient setting. Potential trial patients will be identified by local clinical team members and be given a PIS prior to attending a screening visit. All participants must have had a colonoscopy before screening, where possible recorded for central reading. A magnetic resonance enterography (MRE) to stage disease in accordance with European consensus guidelines^17^ is also required but can be performed after trial entry.

**Figure 2:**
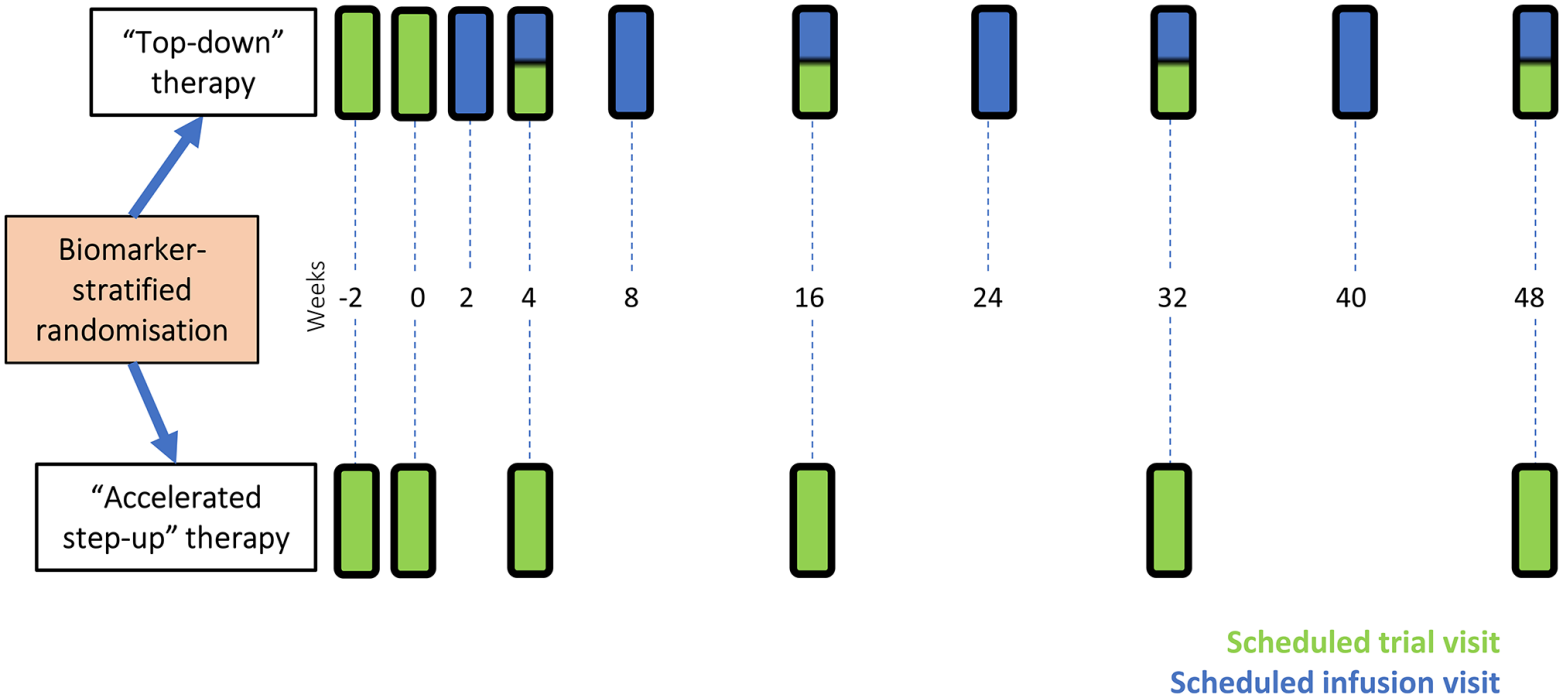
Trial visits for participants. Patients randomised to ‘accelerated step-up’ will have a total of five further trial visits after their initial screening visit. Participants randomised to the ‘top-down’ group will be started on infliximab at week 2. All further infliximab infusion visits should be aligned to scheduled trial visits wherever possible in order to minimise visit burden for participants. Participants in the top-down group will also have five trial visits and will also attend hospital an additional four times for infliximab infusions. Randomisation occurs at week 0.

Assessments, data collection and obtaining informed consent will be performed by appropriately trained research staff, as delegated by the principal investigator at each site. At trial visits, clinical data will be collected as well as samples for local and central processing—collection, evaluation and storage of these samples are outlined in the full trial protocol. Participants receiving infliximab should have infusion visits aligned with trial visits, as shown in figure 2, to reduce visit burden and the placebo effect associated with extra visits.^18^ Following their final trial visit, participants will return to normal standard of care, according to local clinical practice.

Only adverse events (AEs) that relate to CD, drug therapy for CD (sufficiently severe to require a change of treatment), or the biomarker sample collection will be recorded and assessed. Safety reporting and assessment of causality and expectedness of serious AEs will occur within standard timelines. The trial sponsors will arrange insurance for negligent harm caused as a result of protocol design and for non-negligent harm arising through participation in the clinical trial.

#### Sample size calculation

We will recruit 400 participants into the PROFILE trial. This sample size was determined using a power calculation in which power was calculated by simulating 10 000 study designs and counting how many times a significant result was obtained. This was based on previously published remission rates for the primary endpoint,^3 7^ the observed ratio of the IBD^hi^/IBD^lo^ biomarker result in existing cohorts (1:1) and the observed remission rates in each of these cohorts.^14^

#### Statistical procedures and data analysis plan

The primary analysis is powered as an interaction analysis, where the interaction refers to the difference between the relative benefit of top-down over accelerated step-up in each subgroup. This analytical strategy maximises the information available from each subgroup, and will determine whether the biomarker can accurately match patients to the most appropriate treatment strategy. Assuming an interaction of 0.3, a sample size of 346 will provide 90% power (estimated with 95% CIs and tested at a two-tailed, 5% significance level). To allow for a~13.5% drop out rate, 400 participants will be recruited across approximately 50 sites. This will require recruitment of ~4 participants per site per year, which is a rate consistent with previous recruitment to investigator-led IBD studies in the UK.^19^ Recruitment began in December 2017.

To control for multiple testing, we will perform a closed testing procedure over the primary and six secondary endpoints, testing the biomarker–treatment interaction. A well-described methodology combining gate-keeping and Holm-Bonferroni methods in formal hypothesis testing will be used,^20^ as outlined in online supplementary figure 1. The secondary outcome measures will include an endoscopic assessment of mucosal healing (in addition to further analyses using MRE data), a quality-of-life assessment and a third outcome measure related to overall burden of disease (this hierarchically includes number of flares, cumulative steroid exposure, number of hospital admissions and number of Crohn’s-related surgeries).

10.1136/bmjopen-2018-026767.supp1 Supplementary file 1

Mucosal healing has been associated with improved long-term outcomes in CD.^21 22^ The use of central reading, in which the endoscopic images or video recordings are externally evaluated, has been further associated with a reduction in placebo response rates,^23^ in part due to more stringent application of inclusion criteria and assessment of endoscopic response.^24^ The PROFILE trial will use video recording of colonoscopy at the end of the trial period in all patients and at the outset in as many patients as possible, using the SES-CD,^25^ a scoring tool that has been shown to have high inter-rater and intrarater reliability.^26^ To date, many trials using endoscopic endpoints have applied *post hoc* analyses in small cohorts, resulting in limited power to detect effects.^27^ In this respect, the PROFILE trial will be one of the largest trials to analyse mucosal healing routinely and the first to do so in the setting of adults with CD treated with top-down therapy from diagnosis.

An MRE will be performed at the end of the trial period in all patients. There is increasing interest in the use of MRE as a measure of disease activity in clinical trials, with the development of imaging scores such as the Magnetic Resonance Index of Activity (MaRIA).^28 29^ This, and other similar scores, have often been validated and refined in relatively small cohorts^30^ and none are in routine clinical use. With 400 participants, the PROFILE trial will enable further evaluation of the MaRIA score both in terms of confirming treatment response and as an evaluative index.^31^

Quality-of-life assessments will be performed over repeated visits and will be analysed using a mixed effect repeat measure analysis with a clustered patient-level residual error with unstructured covariance over visits, fixed effects for visit and all other covariates assumed to have a constant fixed effect over time.

It is anticipated that future data collection will also take place following completion of treatment to assess disease burden and the longer term impact of top-down versus accelerated step-up treatment approaches on subsequent disease course for these patients.

## Conclusions

Currently, there is a clear unmet need in the management of IBD, in that treatment strategies—whatever they may be—are typically applied in a one-size-fits-all manner or using ‘prognostic’ markers that have not been shown to be able to guide therapy.

The PROFILE trial is the first biomarker-stratified trial in IBD and will investigate whether a blood-based biomarker, assessed at diagnosis, can stratify patients with CD to receive therapy that is appropriately matched to their subsequent disease course.

If stratification by IBD^hi^/IBD^lo^ status is demonstrated to improve clinical outcomes by appropriately identifying those patients who require top-down therapy and those who can be safely managed with accelerated step-up therapy, this would represent a step change in the management of CD and would help make personalised medicine a reality for patients.

### Ethics and dissemination

Recruitment for the PROFILE trial began in December 2017 and is currently ongoing at sites around the United Kingdom. On completion of the trial, the data will be analysed and tabulated and a final trial report prepared. Following trial completion and analysis, the results will be presented at scientific meetings and submitted for publication in a peer-reviewed journal. Press releases will be prepared to accompany publication of this trial in order to share the results more widely with the global medical community, trial participants and patient support groups. Reasonable applications for individual clinical trial participant-level data will be considered by the trial team and shared on a controlled access basis if approved. Authorship of final trial outputs will be assigned in accordance with guidelines set out by the International Committee of Medical Journal Editors. The Standard Protocol Items: Recommendations for Interventional Trials reporting guidelines have been used in preparation of this article.^32^

### Patient records

Data are collected via a paper case report form, provided by the trial coordination team, and after being input electronically, will be stored in a secured database. Participants will only be identifiable by a trial-specific number in the database. Essential documents will be retained until at least 15 years after the publication of the clinical trial report.

## Trial committees

The unblinded data will be presented to the Data Monitoring Committee, who will meet on a regular basis throughout the trial and who are independent from the sponsor. The Data Monitoring Committee will then prepare a report for the Trial Steering Committee who will provide overall supervision of the trial.

## Supplementary Material

Reviewer comments

Author's manuscript
