# Supplementary material for: PRedicting Outcomes For Crohn’s dIsease using a moLecular biomarkEr (PROFILE): protocol for a multicentre, randomised, biomarker-stratified trial
Source: BMJ Open. 2018 Dec 5;8(12):e026767. doi: 10.1136/bmjopen-2018-026767 (PMC6286485; doi:10.1136/bmjopen-2018-026767)
Supplement: Supplementary file 1 [file bmjopen-2018-026767supp001.pdf]

Supplementary Material

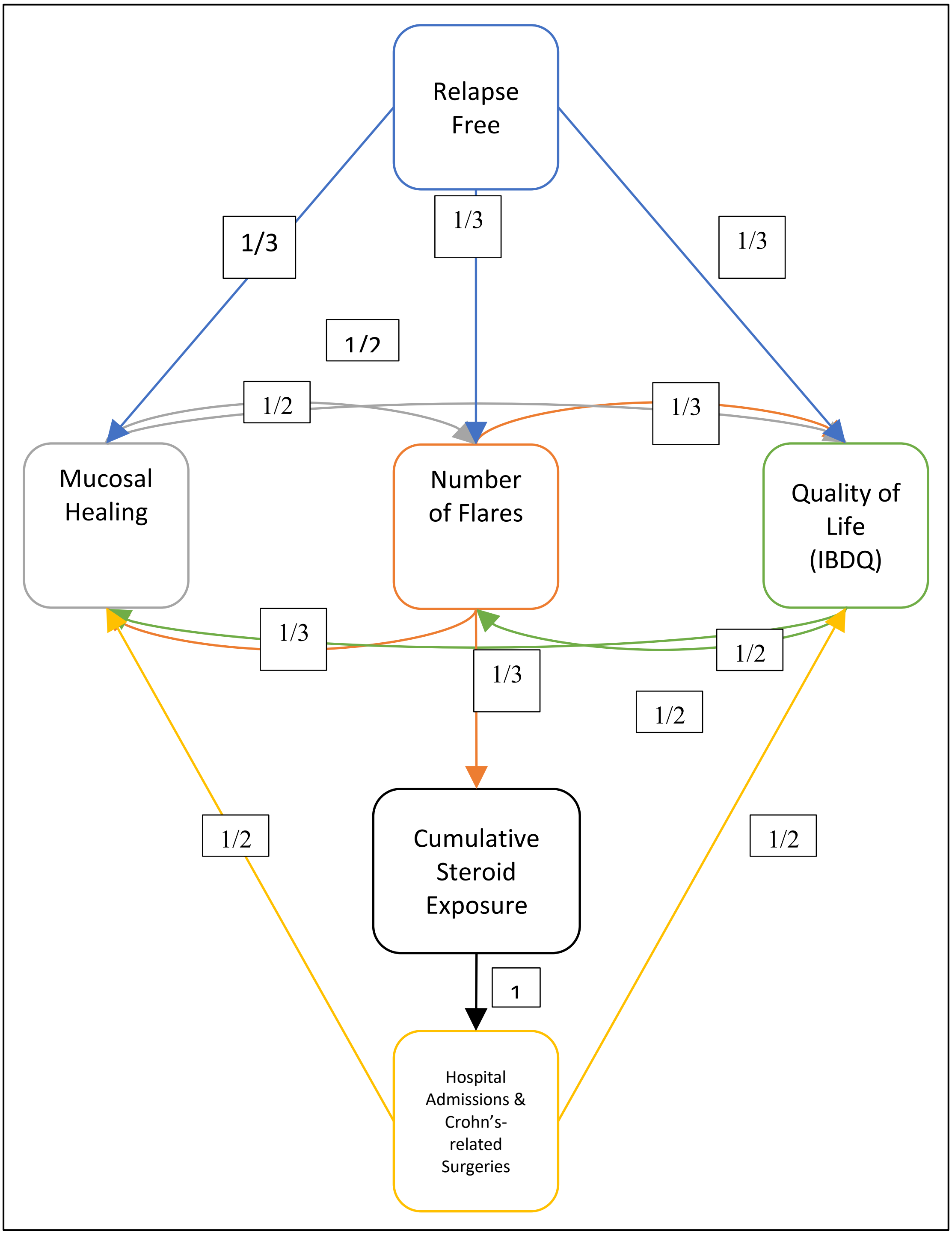

Supplementary Figure 1 – Statistical approach for the PROFILE trial.

The methodology will combine together gate-keeping and Holm-Bonferroni methods in formal hypothesis testing, with the above diagram defining how the significance levels will be transitioned assuming an initial configuration of 5% at the primary endpoint (relapse-free remission) and 0% on all other tests. All the secondary endpoints are continuous variables and will be analysed using a linear regression framework adjusting for baseline covariates.
