# Supplementary material for: PRedicting Outcomes For Crohn’s dIsease using a moLecular biomarkEr (PROFILE): protocol for a multicentre, randomised, biomarker-stratified trial
Source: BMJ Open. 2018 Dec 5;8(12):e026767. doi: 10.1136/bmjopen-2018-026767 (PMC6286485; doi:10.1136/bmjopen-2018-026767)
Supplement: Author's manuscript [file bmjopen-2018-026767.draft_revisions.pdf]

BMJ Open is committed to open peer review. As part of this commitment we make the peer review history of every article we publish publicly available.

When an article is published we post the peer reviewers' comments and the authors' responses online. We also post the versions of the paper that were used during peer review. These are the versions that the peer review comments apply to.

The versions of the paper that follow are the versions that were submitted during the peer review process. They are not the versions of record or the final published versions. They should not be cited or distributed as the published version of this manuscript.

BMJ Open is an open access journal and the full, final, typeset and author-corrected version of record of the manuscript is available on our site with no access controls, subscription charges or pay-per-view fees (http://bmjopen.bmj.com).

If you have any questions on BMJ Open's open peer review process please email info.bmjopen@bmj.com

BMJ Open

Predicting outcomes for Crohn's disease using a molecular biomarker (PROFILE): protocol for a multi-centre, randomised, biomarker-stratified trial

Journal:	*BMJ Open*
Manuscript ID	bmjopen-2018-026767
Article Type:	Protocol
Date Submitted by the Author:	19-Sep-2018

Complete List of Authors:	Parkes, M; University of Cambridge, Department of Medicine<br>Noor, Nurulamin; University of Cambridge, Department of Medicine<br>Dowling, Francis; Cambridge Clinical Trials Unit<br>Leung, Harvey; Cambridge Clinical Trials Unit<br>Bond, Simon; Cambridge Clinical Trials Unit; MRC Biostatistics Unit<br>Whitehead, Lynne; Cambridge University Hospitals NHS Foundation Trust, Clinical Trials Pharmacy<br>Upponi, Sara; Cambridge University Hospitals NHS Foundation Trust, Department of Radiology<br>Kinnon, Paul; PredictImmune Ltd<br>Sandham, Andrew; PredictImmune Ltd<br>Lyons, Paul; University of Cambridge, Department of Medicine; PredictImmune Ltd<br>McKinney, Eoin; University of Cambridge, Department of Medicine; PredictImmune Ltd<br>Smith, Kenneth; University of Cambridge, Department of Medicine; PredictImmune Ltd<br>Lee, James; University of Cambridge, Department of Medicine

Keywords:	GENETICS, Inflammatory bowel disease < GASTROENTEROLOGY, Immunology < BASIC SCIENCES, Gastroenterology < INTERNAL MEDICINE, Clinical trials < THERAPEUTICS

SCHOLARONE™
Manuscripts

Predicting outcomes for Crohn's disease using a molecular biomarker (PROFILE): protocol for a multi-centre, randomised, biomarker-stratified trial

Authors

Miles Parkes[1*†], Nurulamin M Noor[1*], Francis Dowling[2], Harvey Leung[2], Simon Bond[2,3], Lynne Whitehead[4], Sara Upponi[5], Paul Kinnon[6], Andrew P Sandham[6], Paul A Lyons[1,6], Eoin F McKinney[1,6], Kenneth GC Smith[1,6], James C Lee[1†]

1. Department of Medicine, University of Cambridge School of Clinical Medicine, Addenbrooke's Hospital, Cambridge, United Kingdom.

2. Cambridge Clinical Trials Unit, Coton House, Level 6, Cambridge University Hospitals NHS Foundation Trust, Cambridge, United Kingdom.

3. Medical Research Council Biostatistics Unit, Forvie Site, Robinson Way, Cambridge, United Kingdom.

4. Clinical Trials Pharmacy, Addenbrooke's Hospital, Cambridge, United Kingdom.

5. Department of Radiology, Addenbrooke's Hospital, Cambridge, United Kingdom.

6. PredictImmune Ltd., Maia Building 270, Babraham Research Campus, Cambridge, United Kingdom.

* These authors contributed equally
† Corresponding authors (MP: miles.parkes@addenbrookes.nhs.uk, JCL: jcl65@cam.ac.uk)

For peer review only

ABSTRACT

Background

The course of Crohn's disease (CD) varies substantially between individuals, but reliable prognostic markers do not exist. This hinders disease management because patients with aggressive disease are undertreated by conventional "Step-Up" therapy (in which treatment is gradually escalated in response to refractory or relapsing disease) while those with more indolent disease would be exposed to unnecessary treatment-related toxicity if a more aggressive "Top-Down" approach were indiscriminately used. PROFILE will assess whether a prognostic transcriptional biomarker, that we have developed and validated, can improve clinical outcomes by facilitating personalized therapy in CD. This represents the first the biomarker-stratified trial in inflammatory bowel disease.

Methods and analysis

This biomarker-stratified trial will compare the relative efficacy of "Top-Down" and "Accelerated Step-Up" therapy between biomarker-defined subgroups of patients with newly-diagnosed CD. 400 participants from ~50 UK centres will be recruited. Subjects within each biomarker subgroup (IBD^{hi} or IBD^{lo}) will be randomised (1:1) to receive one of the treatment strategies until trial completion (48 weeks). The primary outcome is the incidence of sustained surgery and steroid-free remission from completion of induction treatment through to week 48. Secondary outcomes include mucosal healing, quality of life assessments, and surrogate measures of disease burden including number of flares, cumulative steroid exposure, number of hospital admissions and number of Crohn's-related surgeries (assessed hierarchically). Analyses will compare the relative benefit of the treatment strategies in each biomarker-defined subgroup, powered as an interaction analysis, to determine whether the biomarker can accurately match patients to the most appropriate therapy.

Ethics and dissemination

Ethical approval has been obtained and recruitment is underway at sites around the United Kingdom. Following trial completion and data analysis, the

For peer review only - http://bmjopen.bmj.com/site/about/guidelines.xhtml

results of the trial will be submitted for publication in peer-reviewed journals and presented at international conferences.

Trial registration details: ISRCTN: 11808228 (registered 3/11/2017).

[297/300]

Keywords

Crohn's disease, trial, biomarker, personalised medicine, stratified

For peer review only

For peer review only - http://bmjopen.bmj.com/site/about/guidelines.xhtml

Article Summary

Strengths and limitations of this study

- The first biomarker-stratified trial in inflammatory bowel disease, using a blood-based gene expression assay to stratify newly diagnosed Crohn's disease patients into subgroups (IBD^{hi} or IBD^{lo}) that have been shown to correlate with disease prognosis.
- A multi-centre, randomised trial of 400 patients across approximately 50 sites, comparing "Top-Down" versus "Accelerated Step-Up" treatment approaches in each biomarker-defined subgroup.
- The largest interventional trial ever conducted in adult patients with newly-diagnosed Crohn's disease.
- Pragmatic trial design with wide eligibility criteria, although patient population currently limited to the United Kingdom.
- Findings have the potential to demonstrate that personalised therapy can be effectively delivered to patients with Crohn's disease at the time of diagnosis.

For peer review only

For peer review only - http://bmjopen.bmj.com/site/about/guidelines.xhtml

Introduction

Crohn's disease (CD) is a relapsing-remitting form of inflammatory bowel disease (IBD) that can affect any part of the intestine, most commonly the ileum and/or colon. It is a common condition, affecting ~1 in 400-500 people in Northwestern Europe and North America, with a steadily rising global incidence.[1,2]

Like many other immune-mediated diseases, the course of CD varies substantially between affected individuals, but no reliable prognostic markers currently exist. The most common treatment strategy in CD is therefore based on a reactive, step-wise escalation in therapy that occurs in response to recurrent flares or persistently active disease. This approach (termed "Step-Up") should not over-treat patients but will inevitably expose some individuals to cumulative intestinal damage and disease-related complications while therapies that are insufficiently potent for them are trialled.

In 2008, it was shown that early use of anti-TNFα monoclonal antibodies (anti-TNFα therapy) was superior to conventional "Step-Up" management.[3] Further support for early anti-TNFα use came from registration trials, which demonstrated greater efficacy of anti-TNFα therapy when it was used earlier in the disease course;[4,5] and the SONIC trial, which showed that combining anti-TNFα (Infliximab) with Azathioprine (termed combination or "Top-Down" therapy) achieved results superior to either alone.[6] However, it is widely recognised that the indiscriminate use of combination therapy in all patients would expose those patients destined for mild disease to the risks and side-effects of treatment that their disease did not require, and would also be economically unfeasible.

In an attempt to reconcile these issues, subsequent trials have sought to identify approaches that could still deliver relatively early, aggressive therapy but also be economically feasible. The REACT trial, for example, investigated whether accelerating more quickly up the treatment ladder ("Accelerated Step-Up") would lead to better outcomes.[7] Similarly, the AZTEC and RAPID trials investigated whether initiating Azathioprine, a less potent but cheaper

For peer review only - http://bmjopen.bmj.com/site/about/guidelines.xhtml

immunomodulator, in all patients at diagnosis would improve outcomes.[8,9] However, none of these studies have demonstrated improved efficacy over standard care, leading many to conclude that a "precision" (or "personalised") approach would be required in which the most potent treatments are targeted to those who need them. Unfortunately, despite investigation into the prognostic utility of clinical, genetic and serological markers, there remain no well-validated prognostic tools for CD that can reliably predict the disease course from diagnosis. Indeed, a recent priority setting partnership group, tasked with identifying major areas of unmet need in IBD research, designated the need to develop markers to guide treatment for individual patients as the most important unmet need in IBD.[10] Consistent with this, a survey of 52 US and 50 UK gastroenterologists (commissioned through Apex Healthcare Consulting) showed that nearly all gastroenterologists recognised a need for an assay that could predict the clinical outcome and probability of relapse in CD (UK 98%, US 94%; Table 1). Moreover, if the results of such a biomarker enabled gastroenterologists to amend their treatment approach, all of the respondents would use the test in their practice (Table 1).

Our group has previously identified a gene expression signature in peripheral blood CD8+ T cells from patients with active, untreated IBD (and other autoimmune diseases) that is related to T cell exhaustion and which correlates with subsequent prognosis.[11–13] Patients in the IBD1 subgroup, defined by this signature, had a much more aggressive disease than those in the IBD2 subgroup, with earlier recurrence of disease and more flares over time.[11] To help translate this to routine clinical practice, we have since developed a whole blood qPCR assay that can identify patient subgroups which are analogous to those identified by the CD8 signature, but which does not require cell separation (manuscript in preparation). This assay has been independently prospectively validated in a cohort of 84 IBD patients from four centres around the UK.[14] We now propose to conduct a biomarker-stratified trial to determine whether this biomarker can facilitate the delivery of personalised medicine in CD and improve outcomes.

This manuscript summarises the approved PROFILE trial protocol that is in

For peer review only - http://bmjopen.bmj.com/site/about/guidelines.xhtml

use at the time of publication (version 3.0, 30th April 2018). The full version of the protocol is available at: http://www.crohnsprofiletrial.com/index.php/investigators/downloads/.

The PROFILE trial participant information sheet (PIS) that is in use at the time of publication (version 3.1, 25th June 2018) is available at: http://www.crohnsprofiletrial.com/index.php/participants/downloads/.

Any future amendments to this protocol or participant information sheet will require agreement with the Sponsors and amendments will only be initiated following approval by a Research Ethics Committee (REC).

For peer review only - http://bmjopen.bmj.com/site/about/guidelines.xhtml

	UK (n = 50)	US (n = 52)
“CD patients are at moderate-to-high risk of relapse throughout their lives”	Agree – 80% (40)	Agree – 79% (41)
“There is a need for an assay that would predict clinical outcome and probability of relapse in CD”	Agree - 98% (49)	Agree - 94% (49)
Would you use a test to predict clinical outcome and probability of relapse even if you could not change your treatment approach?	Yes – 58% (29)	Yes – 54% (28)
Would you use a test to predict clinical outcome and probability of relapse if it enabled you to alter your treatment approach?	Yes – 100% (50)	Yes – 100% (52)
How many days following a test to predict clinical outcome and probability of relapse would you require the results for this to be useful?	10 days (mean)	9 days (mean)

Table 1. Summary results of an independent 2015 survey of practising gastroenterologists performed by Apex Healthcare Consulting

Gastroenterologists: Clinically active attending physicians (US) or consultants (UK) with 5-30 years specialty experience, including inflammatory bowel disease caseload. Survey funded by Wellcome Trust (Interim Translational Award 099450/Z/12/Z)

For peer review only

For peer review only - http://bmjopen.bmj.com/site/about/guidelines.xhtml

Aims and objectives

The PROFILE trial will test whether stratification using a whole blood gene expression biomarker can facilitate personalised therapy in CD and improve clinical outcomes. The hypothesis is that the biomarker will identify individuals destined to run an aggressive, relapsing course, and that in these individuals a greater benefit of early “Top-Down” therapy will be observed. Similarly, we hypothesise that the biomarker will reliably identify those patients destined to experience more indolent disease, who can be effectively managed using conventional “Accelerated Step-Up” approaches without the risks and side-effects of unnecessary immunosuppression.

In addition, the trial will seek to advance scientific understanding of CD through the collection of a range of biological samples for future exploratory translational and scientific studies. These will include microbial, metabolomic, proteomic, genetic and transcriptomic samples.

Methods and analysis

Trial design and flowchart

The trial is designed as a randomised, biomarker-stratified trial to assess the relative benefit of different treatment approaches in biomarker-defined subgroups. This is an established design for the validation of predictive biomarkers,[15] and has been used widely in the setting of oncology trials.[16] Within each biomarker group, patients will be randomised in a 1:1 ratio to receive either “Top-Down” or Accelerated Step-Up” therapy (Figure 1).

Trial sites

PROFILE is a multi-centre trial based in National Health Service hospitals within the UK. This trial aims to recruit 400 participants with newly diagnosed CD and will be conducted in approximately 50 sites (http://www.crohnsprofiletrial.com/index.php/investigators/).

Trial duration

After providing informed consent, participants will be enrolled within the trial for 48 weeks following the baseline visit. There will be a total of 6 mandatory

For peer review only - http://bmjopen.bmj.com/site/about/guidelines.xhtml

trial visits, during which data will be collected. These will take place at the same timepoints for all participants and have been timed to coincide with Infliximab infusion visits where possible (for those receiving "Top-Down" therapy). The end of the trial will be the last participant's last visit.

Eligibility criteria

Patients will be considered eligible for enrolment if they fulfil all of the inclusion criteria and meet none of the exclusion criteria (Box 1). The target population are patients with newly diagnosed, active CD who are immunomodulator and anti-TNFα treatment naïve.

Patient and Public Involvement

The development and advancement of personalised medicine in CD represents a major goal for both patients and physicians, and was recently named one of the key research priorities in IBD by a priority setting partnership group, which included both patients and other key stakeholders.[10]

A local panel of CD patients at Cambridge University Hospitals NHS Trust was actively involved in the design of the study and development of study documentation, and feedback was also obtained by a broader panel of non-IBD patients convened by the Cambridge Clinical Trials Unit. Patient support groups (Crohn's and Colitis UK) were engaged during the conduct of the trial via invitation to investigator meetings, presentation to patient support groups, and publicity of the trial on their website and social media platforms. Both Crohn's and Colitis UK and trial participants have also contributed to the content of the trial website (http://www.crohnsprofiletrial.com/index.php/participants), although patients were not directy involved in the recruitment to, or conduct of, the trial.

Following trial completion and reporting, results of the trial will be disseminated in an easy-to-understand format to all trial participants and to Crohn's and Colitis UK, as well to the general public via press releases and the public engagement team at the University of Cambridge.

For peer review only - http://bmjopen.bmj.com/site/about/guidelines.xhtml

Box 1 – Eligibility criteria for the PROFILE trial

Inclusion criteria

Subjects meeting all of the criteria below may be included in the trial:

- CD diagnosed within 3 months using standard endoscopic, histologic or radiological criteria*.
- Clinical evidence of active CD (corresponding to Harvey Bradshaw Index $\geq$ 7).
- Endoscopic evidence of at least moderately active CD (corresponding to SES-CD > 6 or > 4 if limited to the terminal ileum).
- C-reactive protein (CRP) > upper limit of normal on local assay or faecal calprotectin > 200 µg/g.
- Immunomodulator and anti-TNFα treatment naïve**.
- Aged 16-80 years old.

* Newly-diagnosed patchy colonic inflammation, initially diagnosed as indeterminate colitis, would meet inclusion criteria if clinical impression consistent with CD.

** Patients need to have discontinued systemic corticosteroids for one week or more prior to screening assessments and still have ongoing, active disease.

Exclusion criteria

The presence of any of the following would preclude patient inclusion:

- Patients with ulcerative colitis.
- Patients with fistulating peri-anal CD or active perianal sepsis.
- Patients with obstructive symptoms and evidence of a fixed stricture on radiology or colonoscopy, which suggest that the subject is at high risk of requiring surgery over the following year.
- Patients with contra-indications to trial medications.
- Patients who are pregnant or breastfeeding at baseline.
- Other serious medical or psychiatric illness currently ongoing, or experienced in the last 3 months, that could compromise the trial.
- Patients unable to comply with protocol requirements (for reasons including alcohol and/or recreational drug abuse).

For peer review only - http://bmjopen.bmj.com/site/about/guidelines.xhtml

Outcome measures

Primary outcome

Incidence of sustained surgery and steroid-free remission from completion of induction treatment (a standard, 8 week course of oral steroids) through to week 48.*

*remission = Harvey-Bradshaw Index (HBI) $\leq$4. Requirement for a course of systemic glucocorticoids for active CD would result in failure to meet the primary outcome measure.

Secondary outcomes

1. Mucosal healing (assessed using simplified endoscopic score in CD [SES-CD])
2. Quality of life assessment (assessed using Inflammatory Bowel Disease Questionnaire [IBD-Q])
3. Assessment of cumulative disease burden based on:

i. Number of flares by 1 year.
ii. Cumulative glucocorticoid exposure by 1 year.
iii. Steroid-free remission by 1 year.
iv. Number of hospital admissions and CD surgeries by 1 year.

Health economic evaluation

During the course of the trial there will be a local health economic analysis conducted by the Cambridge Centre for Health Services Research, as well as a national health economic analysis conducted by the National Institute for Health and Clinical Excellence (NICE). The findings of these health economic analyses will be disseminated alongside clinical trial findings.

Treatment assignment

All patients considered eligible for the trial at the screening visit will have an 8 week reducing course of Prednisolone initiated for treatment of their active luminal Crohn's disease following screening assessments. Each will be assigned a unique participant ID number, for which a biomarker result will be returned. Anonymised data on all participants who are approached will be

For peer review only - http://bmjopen.bmj.com/site/about/guidelines.xhtml

For peer review only

collated in accordance with Consolidated Standards of Reporting Trials (CONSORT) guidelines. Following biomarker assessment, participants in each biomarker subgroup will be randomly assigned (1:1) to either "Top-Down" or "Accelerated Step-Up" therapy, using a computer-generated algorithm (Figure 1). This will occur within 14 days of screening (plus or minus 5 days).

As the trial is testing the ability of the biomarker to stratify therapy, rather than the efficacy of the individual medications (which are established treatments for CD), PROFILE has been designated a non-CTIMP trial (i.e. not a Clinical Trial of Investigational Medicine Product). All treatments will be open-label, but clinicians and participants will be blinded to biomarker subgroup designation.

Treatment arms

Following induction treatment with Prednisolone, patients will follow the treatment strategy to which they are randomised. These are:

<u>"Accelerated Step-Up" therapy</u>

- Flare 1 (after induction therapy or if disease re-flares during induction therapy): Commence Azathioprine (2.5 mg/kg) OR low dose 6-Mercaptopurine with Allopurinol (if mild intolerance to azathioprine) OR Methotrexate (if severe intolerance to thiopurines or thiopurine methyltransferase [TPMT] null) together with a 12-week reducing course of Prednisolone.
- Flare 2: Commence Infliximab. If sub-optimal response, then for Infliximab dose-escalation as outlined in the full trial protocol.
- Flare 3+ (i.e. disease flare after Infliximab dose optimisation): 8 week reducing course of Prednisolone.

<u>"Top-Down" therapy</u>

- Infliximab started 2 weeks after randomisation with Azathioprine (2.5mg/kg) or alternative immunomodulator as described above. If sub-optimal response, then for Infliximab dose-escalation as described in

For peer review only - http://bmjopen.bmj.com/site/about/guidelines.xhtml

the full trial protocol. The rate of weaning of Prednisolone should be accelerated once Infliximab is commenced to 10mg/week.

- Subsequent disease flares (i.e. disease flare after Infliximab dose optimisation): 8 week reducing course of Prednisolone.

Participants with persistent non-response to Infliximab can have early treatment termination and revert back to standard care, at the discretion of their local clinical team.

Trial procedures & assessments

Newly-diagnosed patients with CD will be recruited from a predominantly outpatient setting. Potential trial patients will be identified by local clinical team members and be given a PIS prior to attending a screening visit. All participants must have had a colonoscopy before screening, where possible recorded for central reading. A Magnetic Resonance Enterography (MRE) to stage disease in accordance with European consensus guidelines[17] is also required but can be performed after trial entry.

Assessments, data collection and obtaining informed consent will be performed by appropriately trained research staff, as delegated by the Principal Investigator at each site. At trial visits, clinical data will be collected as well as samples for local and central processing – collection, evaluation and storage of these samples is outlined in the full trial protocol. Participants receiving Infliximab should have infusion visits aligned with trial visits, as shown in Figure 2, to reduce visit burden and the placebo effect associated with extra visits.[18] Following their final trial visit, participants will return to normal standard of care, according to local clinical practice.

Only adverse events (AEs) that relate to CD, drug therapy for CD (sufficiently severe to require a change of treatment), or the biomarker sample collection will be recorded and assessed. Safety reporting and assessment of causality and expectedness of serious AEs (SAEs) will occur within standard timelines. The trial sponsors will arrange insurance for negligent harm caused as a result of protocol design and for non-negligent harm arising through

For peer review only - http://bmjopen.bmj.com/site/about/guidelines.xhtml

participation in the clinical trial.

Sample size calculation

We will recruit 400 participants into the PROFILE trial. This sample size was determined using a power calculation in which power was calculated by simulating 10,000 study designs and counting how many times a significant result was obtained. This was based on previously published remission rates for the primary endpoint,[3,7] the observed ratio of the IBD^{hi}/IBD^{lo} biomarker result in existing cohorts (1:1), and the observed remission rates in each of these cohorts.[14]

Statistical procedures and data analysis plan

The primary analysis is powered as an interaction analysis, where the interaction refers to the difference between the relative benefit of "Top-Down" over "Accelerated Step-Up" in each subgroup. This analytical strategy maximises the information available from each subgroup, and will determine whether the biomarker can accurately match patients to the most appropriate treatment strategy. Assuming an interaction of 0.3, a sample size of 346 will provide 90% power (estimated with 95% confidence intervals and tested at a 2-tailed, 5% significance level). To allow for a ~13.5% drop out rate, 400 participants will be recruited across approximately 50 sites. This will require recruitment of ~4 participants per site per year, which is a rate consistent with previous recruitment to Investigator-led IBD studies in the UK.[19] Recruitment began in December 2017.

To control for multiple testing, we will perform a closed testing procedure over the primary and 6 secondary endpoints, testing the biomarker-treatment interaction. A well-described methodology combining gate-keeping and Holm-Bonferroni methods in formal hypothesis testing will be used,[20] as outlined in Supplementary Figure 1. The secondary outcome measures will include an endoscopic assessment of mucosal healing (in addition to further analyses using MRE data), a quality of life assessment and a third outcome measure related to overall burden of disease (this hierarchically includes number of flares, cumulative steroid exposure, number of hospital admissions and

For peer review only - http://bmjopen.bmj.com/site/about/guidelines.xhtml

number of Crohn’s-related surgeries).

Mucosal healing has been associated with improved long-term outcomes in CD.[21,22] The use of central reading, in which the endoscopic images or video recordings are externally evaluated, has been further associated with a reduction in placebo response rates,[23] in part due to more stringent application of inclusion criteria and assessment of endoscopic response.[24] The PROFILE trial will utilise video recording of colonoscopy at the end of the trial period in all patients and at the outset in as many patients as possible, using the SES-CD,[25] a scoring tool that has been shown to have high inter- and intra-rater reliability.[26] To date, many trials using endoscopic endpoints have applied *post-hoc* analyses in small cohorts, resulting in limited power to detect effects.[27] In this respect, the PROFILE trial will be one of the largest trials to analyse mucosal healing routinely and the first to do so in the setting of adults with CD treated with “Top-Down” therapy from diagnosis.

An MRE will be performed at the end of the trial period in all patients. There is increasing interest in the use of MRE as a measure of disease activity in clinical trials, with the development of imaging scores such as the Magnetic Resonance Index of Activity (MaRIA).[28,29] This, and other similar scores, have often been validated and refined in relatively small cohorts [30] and none are in routine clinical use. With 400 participants, the PROFILE trial will enable further evaluation of the MaRIA score both in terms of confirming treatment response and as an evaluative index.[31]

Quality of life assessments will be performed over repeated visits and will be analysed using a mixed effect repeat measure analysis with a clustered patient-level residual error with unstructured covariance over visits, fixed effects for visit, and all other covariates assumed to have a constant fixed effect over time.

It is anticipated that future data collection will also take place following completion of treatment to assess disease burden and the longer-term impact

For peer review only - http://bmjopen.bmj.com/site/about/guidelines.xhtml

of “Top-Down” vs. “Accelerated Step-Up” treatment approaches on subsequent disease course for these patients.

Conclusions

Currently there is a clear unmet need in the management of IBD, in that treatment strategies – whatever they may be – are typically applied in a one-size-fits-all manner or using “prognostic” markers that have not been shown to be able to guide therapy.

The PROFILE trial is the first biomarker-stratified trial in IBD and will investigate whether a blood-based biomarker, assessed at diagnosis, can stratify patients with CD to receive therapy that is appropriately matched to their subsequent disease course.

If stratification by IBD^{hi}/IBD^{lo} status is demonstrated to improve clinical outcomes by appropriately identifying those patients who require “Top-Down” therapy and those who can be safely managed with “Accelerated Step-Up” therapy, this would represent a step-change in the management of CD and would help make personalised medicine a reality for patients.

Ethics and dissemination

The trial protocol was approved by the East of England - Cambridge South Research Ethics Committee (Ref: 17/EE/03/82). Recruitment for the PROFILE trial began in December 2017 and is currently ongoing at sites around the United Kingdom. On completion of the trial, the data will be analysed and tabulated and a final trial report prepared. Following trial completion and analysis, the results will be presented at scientific meetings and submitted for publication in a peer-reviewed journal. Press releases will be prepared to accompany publication of this trial in order to share the results more widely with the global medical community, trial participants and patient support groups. Reasonable applications for individual clinical trial participant-level data will be considered by the trial team and shared on a controlled access basis if approved. Authorship of final trial outputs will be assigned in

For peer review only - http://bmjopen.bmj.com/site/about/guidelines.xhtml

accordance with guidelines set out by the International Committee of Medical Journal Editors. The SPIRIT reporting guidelines have been used in preparation of this article.[32]

For peer review only

For peer review only - http://bmjopen.bmj.com/site/about/guidelines.xhtml

References

1 Kaplan GG, Ng SC. Understanding and Preventing the Global Increase of Inflammatory Bowel Disease. *Gastroenterology* 2017;**152**:313–321.e2. doi:10.1053/j.gastro.2016.10.020

2 Ng SC, Shi HY, Hamidi N, *et al.* Worldwide incidence and prevalence of inflammatory bowel disease in the 21st century: a systematic review of population-based studies. *Lancet* 2017;**390**:2769–78. doi:10.1016/S0140-6736(17)32448-0

3 D'Haens G, Baert F, van Assche G, *et al.* Early combined immunosuppression or conventional management in patients with newly diagnosed Crohn's disease: an open randomised trial. *Lancet* 2008;**371**:660–7. doi:10.1016/S0140-6736(08)60304-9

4 Schreiber S, Reinisch W, Colombel JF, *et al.* Subgroup analysis of the placebo-controlled CHARM trial: Increased remission rates through 3years for adalimumab-treated patients with early Crohn's disease. *J Crohn's Colitis* 2013;**7**:213–21. doi:10.1016/j.crohns.2012.05.015

5 Hanauer SB, Feagan BG, Lichtenstein GR, *et al.* Maintenance infliximab for Crohn's disease: The ACCENT I randomised trial. *Lancet* 2002;**359**:1541–9. doi:10.1016/S0140-6736(02)08512-4

6 Colombel JF, Sandborn WJ, Reinisch W, *et al.* Infliximab, azathioprine, or combination therapy for Crohn's disease. *N Engl J Med* 2010;**362**:1383–95. doi:10.1056/NEJMoa0904492

7 Khanna R, Bressler B, Levesque BG, *et al.* Early combined immunosuppression for the management of Crohn's disease (REACT): A cluster randomised controlled trial. *Lancet* 2015;**386**:1825–34. doi:10.1016/S0140-6736(15)00068-9

8 Sans M, Román AL, Esteve M, *et al.* Early Use of Azathioprine Has a Steroid Sparing Effect on Recently Diagnosed Crohn's Disease Patients. *Gastroenterology* 2011;**140**:S-109. doi:10.1016/S0016-5085(11)60441-1

9 Cosnes J, Bourrier A, Laharie D, *et al.* Early administration of azathioprine vs conventional management of Crohn's Disease: a randomized controlled trial. *Gastroenterology* 2013;**145**:758–65.

For peer review only - http://bmjopen.bmj.com/site/about/guidelines.xhtml

doi:10.1053/j.gastro.2013.04.048
10 Hart AL, Lomer M, Verjee A, *et al.* What are the top 10 research questions in the treatment of inflammatory bowel disease? *J Crohn's Colitis* 2017;**11**:204–11. doi:10.1093/ecco-jcc/jjw144
11 Lee JC, Lyons PA, McKinney EF, *et al.* Gene expression profiling of CD8+ T cells predicts prognosis in patients with Crohn disease and ulcerative colitis. *J Clin Invest* 2011;**121**:4170–9. doi:10.1172/JCI59255DS1
12 McKinney EF, Lyons PA, Carr EJ, *et al.* A CD8+T cell transcription signature predicts prognosis in autoimmune disease. *Nat Med* 2010;**16**:586–91. doi:10.1038/nm.2130
13 McKinney EF, Lee JC, Jayne DRW, *et al.* T-cell exhaustion, co-stimulation and clinical outcome in autoimmunity and infection. *Nature* 2015;**523**:612–6. doi:10.1038/nature14468
14 Lee J, Biasci D, Noor N, *et al.* PROFILE trial: predicting outcomes for Crohn's disease using a molecular biomarker. *Gut* 2017;**66**:A22–3. doi:10.1136/gutjnl-2017-314472.44
15 Freidlin B, McShane LM, Korn EL. Randomized clinical trials with biomarkers: Design issues. J. Natl. Cancer Inst. 2010;**102**:152–60. doi:10.1093/jnci/djp477
16 Potti A, Mukherjee S, Petersen R, *et al.* A Genomic Strategy to Refine Prognosis in Early-Stage Non–Small-Cell Lung Cancer. *N Engl J Med* 2006;**355**:570–80. doi:10.1056/NEJMoa060467
17 Gomollón F, Dignass A, Annese V, *et al.* 3rd European evidence-based consensus on the diagnosis and management of Crohn's disease 2016: Part 1: Diagnosis and medical management. *J Crohn's Colitis* 2017;**11**:3–25. doi:10.1093/ecco-jcc/jjw168
18 Su C, Lewis JD, Goldberg B, *et al.* A Meta-Analysis of the Placebo Rates of Remission and Response in Clinical Trials of Active Ulcerative Colitis. *Gastroenterology* 2007;**132**:516–26. doi:10.1053/j.gastro.2006.12.037
19 Heap GA, Weedon MN, Bewshea CM, *et al.* HLA-DQA1-HLA-DRB1 variants confer susceptibility to pancreatitis induced by thiopurine immunosuppressants. *Nat Genet* 2014;**46**:1131–4. doi:10.1038/ng.3093

For peer review only - http://bmjopen.bmj.com/site/about/guidelines.xhtml

20 Bretz F, Maurer W, Brannath W, *et al.* A graphical approach to sequentially rejective multiple test procedures. *Stat Med* 2009;**28**:586–604. doi:10.1002/sim.3495

21 Rutgeerts P, Van Assche G, Sandborn WJ, *et al.* Adalimumab induces and maintains mucosal healing in patients with Crohn's disease: Data from the EXTEND trial. *Inflamm Bowel Dis* 2012;**142**:1102–1111. doi:10.1053/j.gastro.2012.01.035

22 Baert F, Moortgat L, Van Assche G, *et al.* Mucosal Healing Predicts Sustained Clinical Remission in Patients With Early-Stage Crohn's Disease. *Gastroenterology* 2010;**138**:463–8. doi:10.1053/j.gastro.2009.09.056

23 Feagan BG, Sandborn WJ, D'Haens G, *et al.* The role of centralized reading of endoscopy in a randomized controlled trial of mesalamine for ulcerative colitis. *Gastroenterology* 2013;**145**. doi:10.1053/j.gastro.2013.03.025

24 Feagan B, Sandborn WJ, Rutgeerts P, *et al.* Performance of Crohn's disease Clinical Trial Endpoints based upon Different Cutoffs for Patient Reported Outcomes or Endoscopic Activity: Analysis of EXTEND Data. *Inflamm Bowel Dis* 2018;**00**:1–11. doi:10.1093/ibd/izx082

25 Daperno M, D'Haens G, Van Assche G, *et al.* Development and validation of a new, simplified endoscopic activity score for Crohn's disease: The SES-CD. *Gastrointest Endosc* 2004;**60**:505–12. doi:10.1016/S0016-5107(04)01878-4

26 Khanna R, Zou G, D'Haens G, *et al.* Reliability among central readers in the evaluation of endoscopic findings from patients with Crohn's disease. *Gut* 2016;**65**:1119–25. doi:10.1136/gutjnl-2014-308973

27 Reinisch W, Colombel JF, D'Haens G, *et al.* Characterisation of mucosal healing with adalimumab treatment in patients with moderately to severely active Crohn's disease: Results from the EXTEND trial. *J Crohn's Colitis* 2017;**11**:425–34. doi:10.1093/ecco-jcc/jjw178

28 Rimola J, Rodriguez S, García-Bosch O, *et al.* Magnetic resonance for assessment of disease activity and severity in ileocolonic Crohn's disease. *Gut* 2009;**58**:1113–20. doi:10.1136/gut.2008.167957

29 Rimola J, Ordás I, Rodriguez S, *et al.* Magnetic resonance imaging for

For peer review only - http://bmjopen.bmj.com/site/about/guidelines.xhtml

evaluation of Crohn's disease: Validation of parameters of severity and quantitative index of activity. *Inflamm Bowel Dis* 2011;**17**:1759–68. doi:10.1002/ibd.21551

30 Ordás I, Rimola J, Rodríguez S, *et al.* Accuracy of magnetic resonance enterography in assessing response to therapy and mucosal healing in patients with Crohn's disease. *Gastroenterology* 2014;**146**. doi:10.1053/j.gastro.2013.10.055

31 Jairath V, Ordas I, Zou G, *et al.* Reliability of Measuring Ileo-Colonic Disease Activity in Crohn's Disease by Magnetic Resonance Enterography. *Inflamm Bowel Dis* 2018;**24**:440–9. doi:10.1093/ibd/izx040

32 Chan AW, Tetzlaff JM, Altman DG, *et al.* SPIRIT 2013 statement: Defining standard protocol items for clinical trials. Ann. Intern. Med. 2013;**158**:200–7. doi:10.7326/0003-4819-158-3-201302050-00583

For peer review only

For peer review only - http://bmjopen.bmj.com/site/about/guidelines.xhtml

Author statement: MP, NMN, FD, KGCS and JCL wrote the full trial protocol. HL modified final draft versions of the protocol. The Statistics and Data Analysis sections of the trial protocol were written by SB. LW contributed to the Trial Treatment section of the protocol, and SU contributed to the Radiology section. PAL, EFM, MP, KGCS and JCL were the initiators of this trial. All authors took part in reading and final approval of the manuscript.

Acknowledgements: We are grateful for input and assistance from Carrie Bayliss (Cambridge Clinical Trials Unit) and Dr Amanda Wooding (Cambridge Enterprise) in securing funding for this trial, and to Dr Adrian Mander, Prof Robert Gray, Prof Geert D'Haens, Prof Severine Vermeire, Dr Sharon O'Byrne, Prof Subrata Ghosh, Dr Nick Carroll and Dr Ed Godfrey for helpful discussion regarding the protocol. We are grateful to the patients who provided feedback on the study design and documentation. The protocol has been peer reviewed by the British Society of Gastroenterology IBD Clinical Research Group (BSG IBD CRG). Details of participating centres can be found at http://www.crohnsprofiletrial.com/index.php/investigators/. KGCS is a Wellcome Investigator and an NIHR Senior Investigator. EFM and JCL are supported by Wellcome Trust Intermediate Clinical Fellowships (104064/Z/14/Z and 105920/Z/14/Z respectively).

Patient records: Data are collected via a paper CRF, provided by the trial co-ordination team, and after being input electronically, will be stored in a secured database. Participants will only be identifiable by a trial-specific number in the database. Essential documents will be retained until at least 15 years after the publication of the clinical trial report.

Trial committees: The unblinded data will be presented to the Data Monitoring Committee, who will meet on a regular basis throughout the trial and who are independent from the sponsor. The Data Monitoring Committee will then prepare a report for the Trial Steering Committee who will provide overall supervision of the trial.

For peer review only - http://bmjopen.bmj.com/site/about/guidelines.xhtml

Funding: This trial is funded by the Wellcome Trust via an investment in PredictImmune (200448/Z/16/Z). The Apex Healthcare Consulting survey and biomarker development work described herein was funded by a Wellcome Trust Interim Translational Award (099450/Z/12/Z). The trial is co-sponsored by Cambridge University Hospitals NHS Foundation Trust and University of Cambridge. The sponsors are not involved in study design; collection, management, analysis, and interpretation of data; writing of the report; and the decision to submit the report for publication.

Competing interests statement: PAL, EFM, KGCS and JCL are co-inventors on a patent covering the method of assessing prognosis in IBD. PAL, EFM, and KGCS are co-founders and consultants for PredictImmune. PK and APS are employees of PredictImmune. JCL is a consultant for PredictImmune.

Word count: 3281/4000

For peer review only

Figure legends

Figure 1 – Trial Design

Following biomarker stratification, patients will be randomised in a 1:1 fashion to either “Top-Down” or “Accelerated Step-Up” treatment arms.

Figure 2 – Trial visits for participants.

Patients randomised to “Accelerated Step-Up” will have a total of 5 further trial visits after their initial screening visit. Participants randomised to the “Top-Down” group will be started on Infliximab at week 2. All further Infliximab infusion visits should be aligned to scheduled trial visits wherever possible in order to minimise visit burden for participants. Participants in the “Top-Down” group will also have 5 trial visits and will also attend hospital an additional 4 times for infliximab infusions. Randomisation occurs at week 0.

For peer review only

For peer review only - http://bmjopen.bmj.com/site/about/guidelines.xhtml

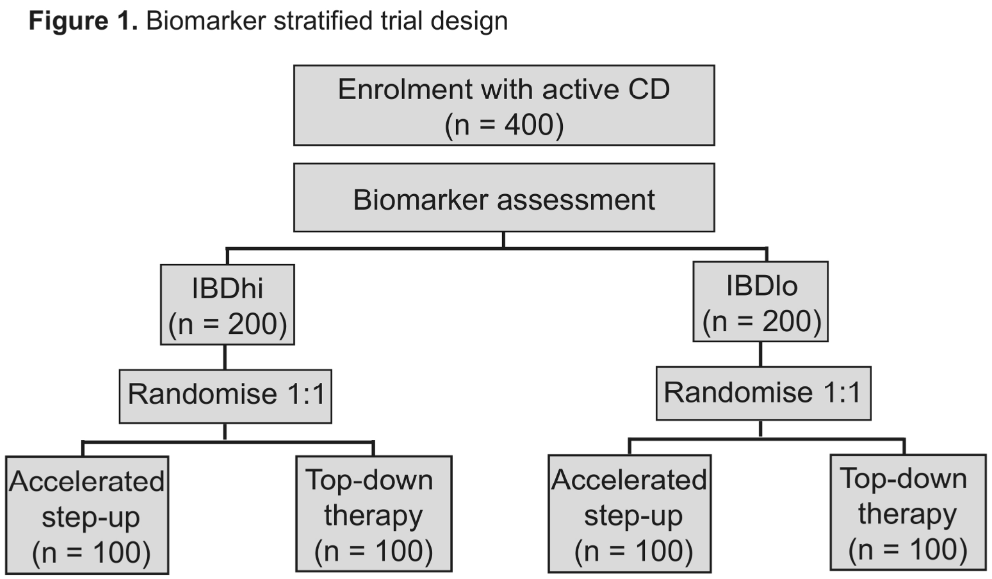

Figure 1 – Trial Design
Following biomarker stratification, patients will be randomised in a 1:1 fashion to either "Top-Down" or "Accelerated Step-Up" treatment arms.

213x124mm (300 x 300 DPI)

view only

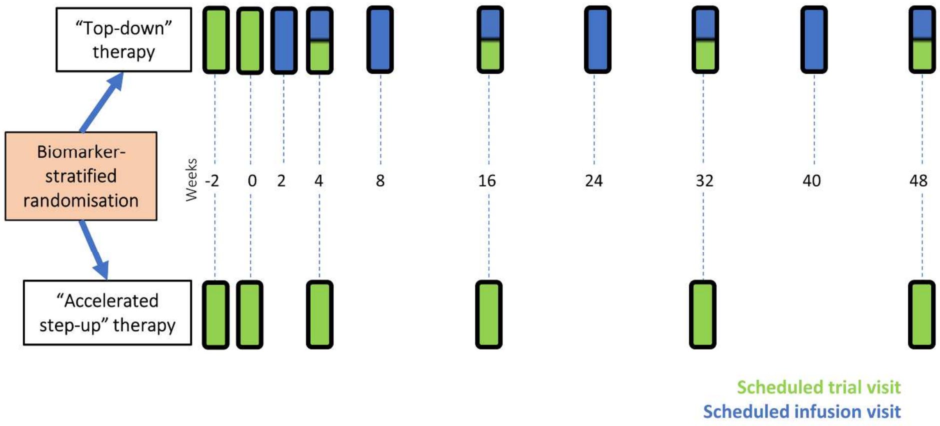

Figure 2 – Trial visits for participants.
Patients randomised to "Accelerated Step-Up" will have a total of 5 further trial visits after their initial screening visit. Participants randomised to the "Top-Down" group will be started on Infliximab at week 2. All further Infliximab infusion visits should be aligned to scheduled trial visits wherever possible in order to minimise visit burden for participants. Participants in the "Top-Down" group will also have 5 trial visits and will also attend hospital an additional 4 times for infliximab infusions. Randomisation occurs at week 0.

125x89mm (600 x 600 DPI)

Supplementary Material

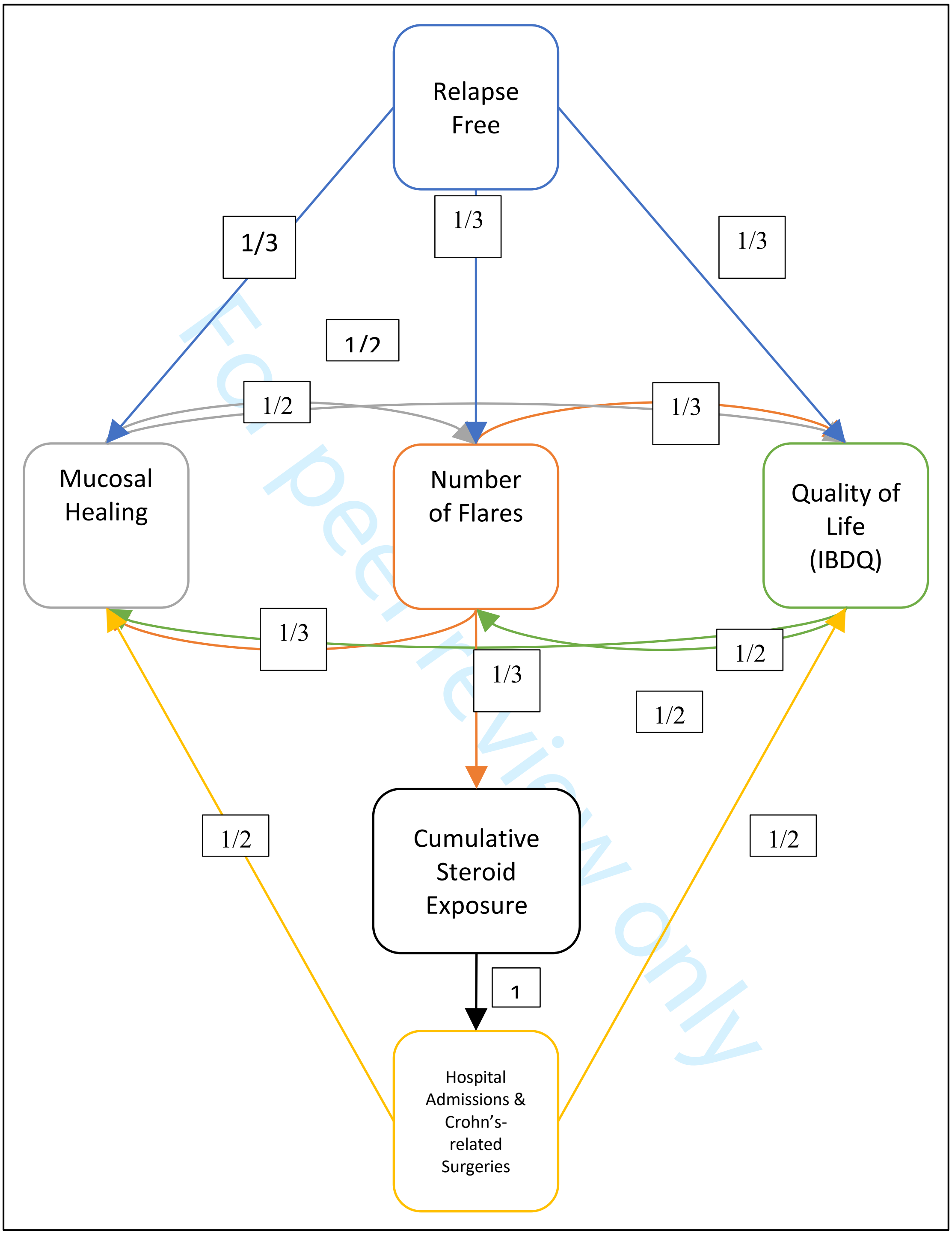

Supplementary Figure 1 – Statistical approach for the PROFILE trial.

The methodology will combine together gate-keeping and Holm-Bonferroni methods in formal hypothesis testing, with the above diagram defining how the significance levels will be transitioned assuming an initial configuration of 5% at the primary endpoint (relapse-free remission) and 0% on all other tests. All the secondary endpoints are continuous variables and will be analysed using a linear regression framework adjusting for baseline covariates.

Reporting checklist for protocol of a clinical trial.

Based on the SPIRIT guidelines.

Instructions to authors

Complete this checklist by entering the page numbers from your manuscript where readers will find each of the items listed below.

Your article may not currently address all the items on the checklist. Please modify your text to include the missing information. If you are certain that an item does not apply, please write "n/a" and provide a short explanation.

Upload your completed checklist as an extra file when you submit to a journal.

In your methods section, say that you used the SPIRIT reporting guidelines, and cite them as:

Chan A-W, Tetzlaff JM, Altman DG, Laupacis A, Gøtzsche PC, Krleža-Jerić K, Hróbjartsson A, Mann H, Dickersin K, Berlin J, Doré C, Parulekar W, Summerskill W, Groves T, Schulz K, Sox H, Rockhold FW, Rennie D, Moher D. SPIRIT 2013 Statement: Defining standard protocol items for clinical trials. Ann Intern Med. 2013;158(3):200-207

		Reporting Item	Page Number
Title	#1	Descriptive title identifying the study design, population, interventions, and, if applicable, trial acronym	1
Trial registration	#2a	Trial identifier and registry name. If not yet registered, name of intended registry	3
Trial registration: data set	#2b	All items from the World Health Organization Trial Registration Data Set	1 (contacts, title), 2 (registration details, countries of recruitment, disease, intervention, study type, sample size, primary and secondary outcomes), 11 (inclusion / exclusion criteria), 17 (ethics review), 24 (sponsor)
Protocol version	#3	Date and version identifier	7

For peer review only

Funding	#4	Sources and types of financial, material, and other support	22-23
Roles and responsibilities: contributorship	#5a	Names, affiliations, and roles of protocol contributors	22
Roles and responsibilities: sponsor contact information	#5b	Name and contact information for the trial sponsor	23
Roles and responsibilities: sponsor and funder	#5c	Role of study sponsor and funders, if any, in study design; collection, management, analysis, and interpretation of data; writing of the report; and the decision to submit the report for publication, including whether they will have ultimate authority over any of these activities	23
Roles and responsibilities: committees	#5d	Composition, roles, and responsibilities of the coordinating centre, steering committee, endpoint adjudication committee, data management team, and other individuals or groups overseeing the trial, if applicable (see Item 21a for data monitoring committee)	22
Background and rationale	#6a	Description of research question and justification for undertaking the trial, including summary of relevant studies (published and unpublished) examining benefits and harms for each intervention	5-7
Background and rationale: choice of comparators	#6b	Explanation for choice of comparators	5-7
Objectives	#7	Specific objectives or hypotheses	9
Trial design	#8	Description of trial design including type of trial (eg, parallel group, crossover,	9

For peer review only

		factorial, single group), allocation ratio, and framework (eg, superiority, equivalence, non-inferiority, exploratory)	
Study setting	#9	Description of study settings (eg, community clinic, academic hospital) and list of countries where data will be collected. Reference to where list of study sites can be obtained	9
Eligibility criteria	#10	Inclusion and exclusion criteria for participants. If applicable, eligibility criteria for study centres and individuals who will perform the interventions (eg, surgeons, psychotherapists)	11
Interventions: description	#11a	Interventions for each group with sufficient detail to allow replication, including how and when they will be administered	13-14
Interventions: modifications	#11b	Criteria for discontinuing or modifying allocated interventions for a given trial participant (eg, drug dose change in response to harms, participant request, or improving / worsening disease)	13-14
Interventions: adherance	#11c	Strategies to improve adherence to intervention protocols, and any procedures for monitoring adherence (eg, drug tablet return; laboratory tests)	14
Interventions: concomitant care	#11d	Relevant concomitant care and interventions that are permitted or prohibited during the trial	13
Outcomes	#12	Primary, secondary, and other outcomes, including the specific measurement variable (eg, systolic blood pressure), analysis metric (eg, change from baseline, final value, time to event), method of aggregation (eg, median, proportion), and time point for each	12

For peer review only

		outcome. Explanation of the clinical relevance of chosen efficacy and harm outcomes is strongly recommended	
Participant timeline	#13	Time schedule of enrolment, interventions (including any run-ins and washouts), assessments, and visits for participants. A schematic diagram is highly recommended (see Figure)	Fig.2
Sample size	#14	Estimated number of participants needed to achieve study objectives and how it was determined, including clinical and statistical assumptions supporting any sample size calculations	9,15
Recruitment	#15	Strategies for achieving adequate participant enrolment to reach target sample size	9
Allocation: sequence generation	#16a	Method of generating the allocation sequence (eg, computer-generated random numbers), and list of any factors for stratification. To reduce predictability of a random sequence, details of any planned restriction (eg, blocking) should be provided in a separate document that is unavailable to those who enrol participants or assign interventions	12-13
Allocation concealment mechanism	#16b	Mechanism of implementing the allocation sequence (eg, central telephone; sequentially numbered, opaque, sealed envelopes), describing any steps to conceal the sequence until interventions are assigned	13
Allocation: implementation	#16c	Who will generate the allocation sequence, who will enrol participants, and who will assign participants to interventions	9,13
Blinding (masking)	#17a	Who will be blinded after assignment to	13

For peer review only

		interventions (eg, trial participants, care providers, outcome assessors, data analysts), and how	
Blinding (masking): emergency unblinding	#17b	If blinded, circumstances under which unblinding is permissible, and procedure for revealing a participant's allocated intervention during the trial	n/a – blinding is to biomarker status not to treatment.
Data collection plan	#18a	Plans for assessment and collection of outcome, baseline, and other trial data, including any related processes to promote data quality (eg, duplicate measurements, training of assessors) and a description of study instruments (eg, questionnaires, laboratory tests) along with their reliability and validity, if known. Reference to where data collection forms can be found, if not in the protocol	22
Data collection plan: retention	#18b	Plans to promote participant retention and complete follow-up, including list of any outcome data to be collected for participants who discontinue or deviate from intervention protocols	22
Data management	#19	Plans for data entry, coding, security, and storage, including any related processes to promote data quality (eg, double data entry; range checks for data values). Reference to where details of data management procedures can be found, if not in the protocol	22
Statistics: outcomes	#20a	Statistical methods for analysing primary and secondary outcomes. Reference to where other details of the statistical analysis plan can be found, if not in the protocol	15, supp. Fig 1
Statistics:	#20b	Methods for any additional analyses (eg,	15-16, supp. Fig 1

For peer review only

additional analyses		subgroup and adjusted analyses)	
Statistics: analysis population and missing data	#20c	Definition of analysis population relating to protocol non-adherence (eg, as randomised analysis), and any statistical methods to handle missing data (eg, multiple imputation)	15-16, supp. Fig 1
Data monitoring: formal committee	#21a	Composition of data monitoring committee (DMC); summary of its role and reporting structure; statement of whether it is independent from the sponsor and competing interests; and reference to where further details about its charter can be found, if not in the protocol. Alternatively, an explanation of why a DMC is not needed	22
Data monitoring: interim analysis	#21b	Description of any interim analyses and stopping guidelines, including who will have access to these interim results and make the final decision to terminate the trial	n/a – no interim analysis is planned.
Harms	#22	Plans for collecting, assessing, reporting, and managing solicited and spontaneously reported adverse events and other unintended effects of trial interventions or trial conduct	14
Auditing	#23	Frequency and procedures for auditing trial conduct, if any, and whether the process will be independent from investigators and the sponsor	n/a - a monitoring plan is currently in the process of being generated - detailing the frequency and scope of monitoring, including of trial conduct. All participating sites will be subject to routine trial specific on-site monitoring.
Research ethics	#24	Plans for seeking research ethics	10

For peer review only

approval		committee / institutional review board (REC / IRB) approval	
Protocol amendments	#25	Plans for communicating important protocol modifications (eg, changes to eligibility criteria, outcomes, analyses) to relevant parties (eg, investigators, REC / IRBs, trial participants, trial registries, journals, regulators)	7
Consent or assent	#26a	Who will obtain informed consent or assent from potential trial participants or authorised surrogates, and how (see Item 32)	14
Consent or assent: ancillary studies	#26b	Additional consent provisions for collection and use of participant data and biological specimens in ancillary studies, if applicable	n/a – no additional consent forms will be used.
Confidentiality	#27	How personal information about potential and enrolled participants will be collected, shared, and maintained in order to protect confidentiality before, during, and after the trial	12-14
Declaration of interests	#28	Financial and other competing interests for principal investigators for the overall trial and each study site	23
Data access	#29	Statement of who will have access to the final trial dataset, and disclosure of contractual agreements that limit such access for investigators	17
Ancillary and post trial care	#30	Provisions, if any, for ancillary and post-trial care, and for compensation to those who suffer harm from trial participation	14
Dissemination policy: trial results	#31a	Plans for investigators and sponsor to communicate trial results to participants, healthcare professionals, the public, and other relevant groups (eg, via publication,	10

For peer review only

		reporting in results databases, or other data sharing arrangements), including any publication restrictions	
Dissemination policy: authorship	#31b	Authorship eligibility guidelines and any intended use of professional writers	17
Dissemination policy: reproducible research	#31c	Plans, if any, for granting public access to the full protocol, participant-level dataset, and statistical code	7 – website link to the full trial protocol.
Informed consent materials	#32	Model consent form and other related documentation given to participants and authorised surrogates	7 – website link to full participant information sheet and consent form.
Biological specimens	#33	Plans for collection, laboratory evaluation, and storage of biological specimens for genetic or molecular analysis in the current trial and for future use in ancillary studies, if applicable	14

The SPIRIT checklist is distributed under the terms of the Creative Commons Attribution License CC-BY-ND 3.0. This checklist can be completed online using https://www.goodreports.org/, a tool made by the EQUATOR Network in collaboration with Penelope.ai

For peer review only

BMJ Open

Predicting outcomes for Crohn's disease using a molecular biomarker (PROFILE): protocol for a multi-centre, randomised, biomarker-stratified trial

Journal:	*BMJ Open*
Manuscript ID	bmjopen-2018-026767.R1
Article Type:	Protocol
Date Submitted by the Author:	08-Oct-2018
Complete List of Authors:	Parkes, M; University of Cambridge, Department of Medicine<br>Noor, Nurulamin; University of Cambridge, Department of Medicine<br>Dowling, Francis; Cambridge Clinical Trials Unit<br>Leung, Harvey; Cambridge Clinical Trials Unit<br>Bond, Simon; Cambridge Clinical Trials Unit; MRC Biostatistics Unit<br>Whitehead, Lynne; Cambridge University Hospitals NHS Foundation Trust, Clinical Trials Pharmacy<br>Upponi, Sara; Cambridge University Hospitals NHS Foundation Trust, Department of Radiology<br>Kinnon, Paul; PredictImmune Ltd<br>Sandham, Andrew; PredictImmune Ltd<br>Lyons, Paul; University of Cambridge, Department of Medicine; PredictImmune Ltd<br>McKinney, Eoin; University of Cambridge, Department of Medicine; PredictImmune Ltd<br>Smith, Kenneth; University of Cambridge, Department of Medicine; PredictImmune Ltd<br>Lee, James; University of Cambridge, Department of Medicine
**Primary Subject Heading**:	Gastroenterology and hepatology
Secondary Subject Heading:	Patient-centred medicine, Genetics and genomics
Keywords:	GENETICS, Inflammatory bowel disease < GASTROENTEROLOGY, Immunology < BASIC SCIENCES, Gastroenterology < INTERNAL MEDICINE, Clinical trials < THERAPEUTICS

SCHOLARONE™
Manuscripts

Predicting outcomes for Crohn's disease using a molecular biomarker (PROFILE): protocol for a multi-centre, randomised, biomarker-stratified trial

Authors

Miles Parkes[1*†], Nurulamin M Noor[1*], Francis Dowling[2], Harvey Leung[2], Simon Bond[2,3], Lynne Whitehead[4], Sara Upponi[5], Paul Kinnon[6], Andrew P Sandham[6], Paul A Lyons[1,6], Eoin F McKinney[1,6], Kenneth GC Smith[1,6], James C Lee[1†]

1. Department of Medicine, University of Cambridge School of Clinical Medicine, Addenbrooke's Hospital, Cambridge, United Kingdom.

2. Cambridge Clinical Trials Unit, Coton House, Level 6, Cambridge University Hospitals NHS Foundation Trust, Cambridge, United Kingdom.

3. Medical Research Council Biostatistics Unit, Forvie Site, Robinson Way, Cambridge, United Kingdom.

4. Clinical Trials Pharmacy, Addenbrooke's Hospital, Cambridge, United Kingdom.

5. Department of Radiology, Addenbrooke's Hospital, Cambridge, United Kingdom.

6. PredictImmune Ltd., Maia Building 270, Babraham Research Campus, Cambridge, United Kingdom.

* These authors contributed equally
† Corresponding authors (MP: miles.parkes@addenbrookes.nhs.uk, JCL: jcl65@cam.ac.uk)

For peer review only

For peer review only - http://bmjopen.bmj.com/site/about/guidelines.xhtml

ABSTRACT

Background

The course of Crohn's disease (CD) varies substantially between individuals, but reliable prognostic markers do not exist. This hinders disease management because patients with aggressive disease are undertreated by conventional "Step-Up" therapy (in which treatment is gradually escalated in response to refractory or relapsing disease) while those with more indolent disease would be exposed to unnecessary treatment-related toxicity if a more aggressive "Top-Down" approach were indiscriminately used. PROFILE will assess whether a prognostic transcriptional biomarker, that we have developed and validated, can improve clinical outcomes by facilitating personalized therapy in CD. This represents the first the biomarker-stratified trial in inflammatory bowel disease.

Methods and analysis

This biomarker-stratified trial will compare the relative efficacy of "Top-Down" and "Accelerated Step-Up" therapy between biomarker-defined subgroups of patients with newly-diagnosed CD. 400 participants from ~50 UK centres will be recruited. Subjects within each biomarker subgroup (IBD^{hi} or IBD^{lo}) will be randomised (1:1) to receive one of the treatment strategies until trial completion (48 weeks). The primary outcome is the incidence of sustained surgery and steroid-free remission from completion of induction treatment through to week 48. Secondary outcomes include mucosal healing, quality of life assessments, and surrogate measures of disease burden including number of flares, cumulative steroid exposure, number of hospital admissions and number of Crohn's-related surgeries (assessed hierarchically). Analyses will compare the relative benefit of the treatment strategies in each biomarker-defined subgroup, powered as an interaction analysis, to determine whether the biomarker can accurately match patients to the most appropriate therapy.

Ethics and dissemination

Ethical approval has been obtained and recruitment is underway at sites around the United Kingdom. Following trial completion and data analysis, the

For peer review only - http://bmjopen.bmj.com/site/about/guidelines.xhtml

results of the trial will be submitted for publication in peer-reviewed journals and presented at international conferences.

Trial registration details: ISRCTN: 11808228 (registered 3/11/2017).

[297/300]

Keywords

Crohn's disease, trial, biomarker, personalised medicine, stratified

For peer review only

For peer review only - http://bmjopen.bmj.com/site/about/guidelines.xhtml

Article Summary

Strengths and limitations of this study

- The first biomarker-stratified trial in inflammatory bowel disease, comparing the relative benefit of “Top-Down” over “Accelerated Step-Up” therapy in biomarker-defined subgroups of patients with newly diagnosed Crohn’s disease.
- The largest interventional trial ever conducted in adult patients with newly-diagnosed Crohn’s disease, incorporating 400 patients across approximately 50 sites.
- Findings have the potential to demonstrate that personalised therapy can be effectively delivered to patients with Crohn’s disease at the time of diagnosis using a blood-based prognostic biomarker.
- Study limited to the United Kingdom.
- Top-down therapy limited to treatment with infliximab and an immunomodulator (which may be superseded by other treatments in the future).

For peer review only

Introduction

Crohn's disease (CD) is a relapsing-remitting form of inflammatory bowel disease (IBD) that can affect any part of the intestine, most commonly the ileum and/or colon. It is a common condition, affecting ~1 in 400-500 people in Northwestern Europe and North America, with a steadily rising global incidence.[1,2]

Like many other immune-mediated diseases, the course of CD varies substantially between affected individuals, but no reliable prognostic markers currently exist. The most common treatment strategy in CD is therefore based on a reactive, step-wise escalation in therapy that occurs in response to recurrent flares or persistently active disease. This approach (termed "Step-Up") should not over-treat patients but will inevitably expose some individuals to cumulative intestinal damage and disease-related complications while therapies that are insufficiently potent for them are trialled.

In 2008, it was shown that early use of anti-TNFα monoclonal antibodies (anti-TNFα therapy) was superior to conventional "Step-Up" management.[3] Further support for early anti-TNFα use came from registration trials, which demonstrated greater efficacy of anti-TNFα therapy when it was used earlier in the disease course;[4,5] and the SONIC trial, which showed that combining anti-TNFα (Infliximab) with Azathioprine (termed combination or "Top-Down" therapy) achieved results superior to either alone.[6] However, it is widely recognised that the indiscriminate use of combination therapy in all patients would expose those patients destined for mild disease to the risks and side-effects of treatment that their disease did not require, and would also be economically unfeasible.

In an attempt to reconcile these issues, subsequent trials have sought to identify approaches that could still deliver relatively early, aggressive therapy but also be economically feasible. The REACT trial, for example, investigated whether accelerating more quickly up the treatment ladder ("Accelerated Step-Up") would lead to better outcomes.[7] Similarly, the AZTEC and RAPID trials investigated whether initiating Azathioprine, a less potent but cheaper

For peer review only - http://bmjopen.bmj.com/site/about/guidelines.xhtml

immunomodulator, in all patients at diagnosis would improve outcomes.[8,9] However, none of these studies have demonstrated improved efficacy over standard care, leading many to conclude that a "precision" (or "personalised") approach would be required in which the most potent treatments are targeted to those who need them. Unfortunately, despite investigation into the prognostic utility of clinical, genetic and serological markers, there remain no well-validated prognostic tools for CD that can reliably predict the disease course from diagnosis. Indeed, a recent priority setting partnership group, tasked with identifying major areas of unmet need in IBD research, designated the need to develop markers to guide treatment for individual patients as the most important unmet need in IBD.[10] Consistent with this, a survey of 52 US and 50 UK gastroenterologists (commissioned through Apex Healthcare Consulting) showed that nearly all gastroenterologists recognised a need for an assay that could predict the clinical outcome and probability of relapse in CD (UK 98%, US 94%; Table 1). Moreover, if the results of such a biomarker enabled gastroenterologists to amend their treatment approach, all of the respondents would use the test in their practice (Table 1).

Our group has previously identified a gene expression signature in peripheral blood CD8+ T cells from patients with active, untreated IBD (and other autoimmune diseases) that is related to T cell exhaustion and which correlates with subsequent prognosis.[11–13] Patients in the IBD1 subgroup, defined by this signature, had a much more aggressive disease than those in the IBD2 subgroup, with earlier recurrence of disease and more flares over time.[11] To help translate this to routine clinical practice, we have since developed a whole blood qPCR assay that can identify patient subgroups which are analogous to those identified by the CD8 signature, but which does not require cell separation (manuscript in preparation). This assay has been independently prospectively validated in a cohort of 84 IBD patients from four centres around the UK.[14] We now propose to conduct a biomarker-stratified trial to determine whether this biomarker can facilitate the delivery of personalised medicine in CD and improve outcomes.

This manuscript summarises the approved PROFILE trial protocol that is in

For peer review only - http://bmjopen.bmj.com/site/about/guidelines.xhtml

use at the time of publication (version 3.0, 30th April 2018). The full version of the protocol is available at: http://www.crohnsprofiletrial.com/index.php/investigators/downloads/.

The PROFILE trial participant information sheet (PIS) that is in use at the time of publication (version 3.1, 25th June 2018) is available at: http://www.crohnsprofiletrial.com/index.php/participants/downloads/.

Any future amendments to this protocol or participant information sheet will require agreement with the Sponsors and amendments will only be initiated following approval by a Research Ethics Committee (REC).

For peer review only

For peer review only - http://bmjopen.bmj.com/site/about/guidelines.xhtml

	UK (n = 50)	US (n = 52)
"CD patients are at moderate-to-high risk of relapse throughout their lives"	Agree – 80% (40)	Agree – 79% (41)
"There is a need for an assay that would predict clinical outcome and probability of relapse in CD"	Agree - 98% (49)	Agree - 94% (49)
Would you use a test to predict clinical outcome and probability of relapse even if you could not change your treatment approach?	Yes – 58% (29)	Yes – 54% (28)
Would you use a test to predict clinical outcome and probability of relapse if it enabled you to alter your treatment approach?	Yes – 100% (50)	Yes – 100% (52)
How many days following a test to predict clinical outcome and probability of relapse would you require the results for this to be useful?	10 days (mean)	9 days (mean)

Table 1. Summary results of an independent 2015 survey of practising gastroenterologists performed by Apex Healthcare Consulting

Gastroenterologists: Clinically active attending physicians (US) or consultants (UK) with 5-30 years specialty experience, including inflammatory bowel disease caseload. Survey funded by Wellcome Trust (Interim Translational Award 099450/Z/12/Z)

For peer review only - http://bmjopen.bmj.com/site/about/guidelines.xhtml

Aims and objectives

The PROFILE trial will test whether stratification using a whole blood gene expression biomarker can facilitate personalised therapy in CD and improve clinical outcomes. The hypothesis is that the biomarker will identify individuals destined to run an aggressive, relapsing course, and that in these individuals a greater benefit of early “Top-Down” therapy will be observed. Similarly, we hypothesise that the biomarker will reliably identify those patients destined to experience more indolent disease, who can be effectively managed using conventional “Accelerated Step-Up” approaches without the risks and side-effects of unnecessary immunosuppression.

In addition, the trial will seek to advance scientific understanding of CD through the collection of a range of biological samples for future exploratory translational and scientific studies. These will include microbial, metabolomic, proteomic, genetic and transcriptomic samples.

Methods and analysis

Trial design and flowchart

The trial is designed as a randomised, biomarker-stratified trial to assess the relative benefit of different treatment approaches in biomarker-defined subgroups. This is an established design for the validation of predictive biomarkers,[15] and has been used widely in the setting of oncology trials.[16] Within each biomarker group, patients will be randomised in a 1:1 ratio to receive either “Top-Down” or Accelerated Step-Up” therapy (Figure 1).

Trial sites

PROFILE is a multi-centre trial based in National Health Service hospitals within the UK. This trial aims to recruit 400 participants with newly diagnosed CD and will be conducted in approximately 50 sites (http://www.crohnsprofiletrial.com/index.php/investigators/).

Trial duration

After providing informed consent, participants will be enrolled within the trial for 48 weeks following the baseline visit. There will be a total of 6 mandatory

For peer review only - http://bmjopen.bmj.com/site/about/guidelines.xhtml

trial visits, during which data will be collected. These will take place at the same timepoints for all participants and have been timed to coincide with Infliximab infusion visits where possible (for those receiving "Top-Down" therapy). The end of the trial will be the last participant's last visit.

Eligibility criteria

Patients will be considered eligible for enrolment if they fulfil all of the inclusion criteria and meet none of the exclusion criteria (Box 1). The target population are patients with newly diagnosed, active CD who are immunomodulator and anti-TNFα treatment naïve.

Patient and Public Involvement

The development and advancement of personalised medicine in CD represents a major goal for both patients and physicians, and was recently named one of the key research priorities in IBD by a priority setting partnership group, which included both patients and other key stakeholders.[10]

A local panel of CD patients at Cambridge University Hospitals NHS Trust was actively involved in the design of the study and development of study documentation, and feedback was also obtained by a broader panel of non-IBD patients convened by the Cambridge Clinical Trials Unit. Patient support groups (Crohn's and Colitis UK) were engaged during the conduct of the trial via invitation to investigator meetings, presentation to patient support groups, and publicity of the trial on their website and social media platforms. Both Crohn's and Colitis UK and trial participants have also contributed to the content of the trial website (http://www.crohnsprofiletrial.com/index.php/participants), although patients were not directy involved in the recruitment to, or conduct of, the trial.

Following trial completion and reporting, results of the trial will be disseminated in an easy-to-understand format to all trial participants and to Crohn's and Colitis UK, as well to the general public via press releases and the public engagement team at the University of Cambridge.

For peer review only - http://bmjopen.bmj.com/site/about/guidelines.xhtml

Box 1 – Eligibility criteria for the PROFILE trial

Inclusion criteria

Subjects meeting all of the criteria below may be included in the trial:

- CD diagnosed within 3 months using standard endoscopic, histologic or radiological criteria*.
- Clinical evidence of active CD (corresponding to Harvey Bradshaw Index $\geq$ 7).
- Endoscopic evidence of at least moderately active CD (corresponding to SES-CD > 6 or > 4 if limited to the terminal ileum).
- C-reactive protein (CRP) > upper limit of normal on local assay or faecal calprotectin > 200 μg/g.
- Immunomodulator and anti-TNFα treatment naïve**.
- Aged 16-80 years old.

* Newly-diagnosed patchy colonic inflammation, initially diagnosed as indeterminate colitis, would meet inclusion criteria if clinical impression consistent with CD.

** Patients need to have discontinued systemic corticosteroids for one week or more prior to screening assessments and still have ongoing, active disease.

Exclusion criteria

The presence of any of the following would preclude patient inclusion:

- Patients with ulcerative colitis.
- Patients with fistulating peri-anal CD or active perianal sepsis.
- Patients with obstructive symptoms and evidence of a fixed stricture on radiology or colonoscopy, which suggest that the subject is at high risk of requiring surgery over the following year.
- Patients with contra-indications to trial medications.
- Patients who are pregnant or breastfeeding at baseline.
- Other serious medical or psychiatric illness currently ongoing, or experienced in the last 3 months, that could compromise the trial.
- Patients unable to comply with protocol requirements (for reasons including alcohol and/or recreational drug abuse).

For peer review only - http://bmjopen.bmj.com/site/about/guidelines.xhtml

Outcome measures

Primary outcome

Incidence of sustained surgery and steroid-free remission from completion of induction treatment (a standard, 8 week course of oral steroids) through to week 48.*

*remission = Harvey-Bradshaw Index (HBI) $\leq$4. Requirement for a course of systemic glucocorticoids for active CD would result in failure to meet the primary outcome measure.

Secondary outcomes

1. Mucosal healing (assessed using simplified endoscopic score in CD [SES-CD])
2. Quality of life assessment (assessed using Inflammatory Bowel Disease Questionnaire [IBD-Q])
3. Assessment of cumulative disease burden based on:

i. Number of flares by 1 year.
ii. Cumulative glucocorticoid exposure by 1 year.
iii. Steroid-free remission by 1 year.
iv. Number of hospital admissions and CD surgeries by 1 year.

Health economic evaluation

During the course of the trial there will be a local health economic analysis conducted by the Cambridge Centre for Health Services Research, as well as a national health economic analysis conducted by the National Institute for Health and Clinical Excellence (NICE). The findings of these health economic analyses will be disseminated alongside clinical trial findings.

Treatment assignment

All patients considered eligible for the trial at the screening visit will have an 8 week reducing course of Prednisolone initiated for treatment of their active luminal Crohn's disease following screening assessments. Each will be assigned a unique participant ID number, for which a biomarker result will be returned. Anonymised data on all participants who are approached will be

For peer review only - http://bmjopen.bmj.com/site/about/guidelines.xhtml

collated in accordance with Consolidated Standards of Reporting Trials (CONSORT) guidelines. Following biomarker assessment, participants in each biomarker subgroup will be randomly assigned (1:1) to either "Top-Down" or "Accelerated Step-Up" therapy, using a computer-generated algorithm (Figure 1). This will occur within 14 days of screening (plus or minus 5 days).

As the trial is testing the ability of the biomarker to stratify therapy, rather than the efficacy of the individual medications (which are established treatments for CD), PROFILE has been designated a non-CTIMP trial (i.e. not a Clinical Trial of Investigational Medicine Product). All treatments will be open-label, but clinicians and participants will be blinded to biomarker subgroup designation.

Treatment arms

Following induction treatment with Prednisolone, patients will follow the treatment strategy to which they are randomised. These are:

"Accelerated Step-Up" therapy

- Flare 1 (after induction therapy or if disease re-flares during induction therapy): Commence Azathioprine (2.5 mg/kg) OR low dose 6-Mercaptopurine with Allopurinol (if mild intolerance to azathioprine) OR Methotrexate (if severe intolerance to thiopurines or thiopurine methyltransferase [TPMT] null) together with a 12-week reducing course of Prednisolone.
- Flare 2: Commence Infliximab. If sub-optimal response, then for Infliximab dose-escalation as outlined in the full trial protocol.
- Flare 3+ (i.e. disease flare after Infliximab dose optimisation): 8 week reducing course of Prednisolone.

"Top-Down" therapy

- Infliximab started 2 weeks after randomisation with Azathioprine (2.5mg/kg) or alternative immunomodulator as described above. If sub-optimal response, then for Infliximab dose-escalation as described in

For peer review only - http://bmjopen.bmj.com/site/about/guidelines.xhtml

the full trial protocol. The rate of weaning of Prednisolone should be accelerated once Infliximab is commenced to 10mg/week.

- Subsequent disease flares (i.e. disease flare after Infliximab dose optimisation): 8 week reducing course of Prednisolone.

Participants with persistent non-response to Infliximab can have early treatment termination and revert back to standard care, at the discretion of their local clinical team.

Trial procedures & assessments

Newly-diagnosed patients with CD will be recruited from a predominantly outpatient setting. Potential trial patients will be identified by local clinical team members and be given a PIS prior to attending a screening visit. All participants must have had a colonoscopy before screening, where possible recorded for central reading. A Magnetic Resonance Enterography (MRE) to stage disease in accordance with European consensus guidelines[17] is also required but can be performed after trial entry.

Assessments, data collection and obtaining informed consent will be performed by appropriately trained research staff, as delegated by the Principal Investigator at each site. At trial visits, clinical data will be collected as well as samples for local and central processing – collection, evaluation and storage of these samples is outlined in the full trial protocol. Participants receiving Infliximab should have infusion visits aligned with trial visits, as shown in Figure 2, to reduce visit burden and the placebo effect associated with extra visits.[18] Following their final trial visit, participants will return to normal standard of care, according to local clinical practice.

Only adverse events (AEs) that relate to CD, drug therapy for CD (sufficiently severe to require a change of treatment), or the biomarker sample collection will be recorded and assessed. Safety reporting and assessment of causality and expectedness of serious AEs (SAEs) will occur within standard timelines. The trial sponsors will arrange insurance for negligent harm caused as a result of protocol design and for non-negligent harm arising through

For peer review only - http://bmjopen.bmj.com/site/about/guidelines.xhtml

participation in the clinical trial.

Sample size calculation

We will recruit 400 participants into the PROFILE trial. This sample size was determined using a power calculation in which power was calculated by simulating 10,000 study designs and counting how many times a significant result was obtained. This was based on previously published remission rates for the primary endpoint,[3,7] the observed ratio of the IBD^{hi}/IBD^{lo} biomarker result in existing cohorts (1:1), and the observed remission rates in each of these cohorts.[14]

Statistical procedures and data analysis plan

The primary analysis is powered as an interaction analysis, where the interaction refers to the difference between the relative benefit of "Top-Down" over "Accelerated Step-Up" in each subgroup. This analytical strategy maximises the information available from each subgroup, and will determine whether the biomarker can accurately match patients to the most appropriate treatment strategy. Assuming an interaction of 0.3, a sample size of 346 will provide 90% power (estimated with 95% confidence intervals and tested at a 2-tailed, 5% significance level). To allow for a ~13.5% drop out rate, 400 participants will be recruited across approximately 50 sites. This will require recruitment of ~4 participants per site per year, which is a rate consistent with previous recruitment to Investigator-led IBD studies in the UK.[19] Recruitment began in December 2017.

To control for multiple testing, we will perform a closed testing procedure over the primary and 6 secondary endpoints, testing the biomarker-treatment interaction. A well-described methodology combining gate-keeping and Holm-Bonferroni methods in formal hypothesis testing will be used,[20] as outlined in Supplementary Figure 1. The secondary outcome measures will include an endoscopic assessment of mucosal healing (in addition to further analyses using MRE data), a quality of life assessment and a third outcome measure related to overall burden of disease (this hierarchically includes number of flares, cumulative steroid exposure, number of hospital admissions and

For peer review only - http://bmjopen.bmj.com/site/about/guidelines.xhtml

number of Crohn's-related surgeries).

Mucosal healing has been associated with improved long-term outcomes in CD.[21,22] The use of central reading, in which the endoscopic images or video recordings are externally evaluated, has been further associated with a reduction in placebo response rates,[23] in part due to more stringent application of inclusion criteria and assessment of endoscopic response.[24] The PROFILE trial will utilise video recording of colonoscopy at the end of the trial period in all patients and at the outset in as many patients as possible, using the SES-CD,[25] a scoring tool that has been shown to have high inter- and intra-rater reliability.[26] To date, many trials using endoscopic endpoints have applied *post-hoc* analyses in small cohorts, resulting in limited power to detect effects.[27] In this respect, the PROFILE trial will be one of the largest trials to analyse mucosal healing routinely and the first to do so in the setting of adults with CD treated with "Top-Down" therapy from diagnosis.

An MRE will be performed at the end of the trial period in all patients. There is increasing interest in the use of MRE as a measure of disease activity in clinical trials, with the development of imaging scores such as the Magnetic Resonance Index of Activity (MaRIA).[28,29] This, and other similar scores, have often been validated and refined in relatively small cohorts [30] and none are in routine clinical use. With 400 participants, the PROFILE trial will enable further evaluation of the MaRIA score both in terms of confirming treatment response and as an evaluative index.[31]

Quality of life assessments will be performed over repeated visits and will be analysed using a mixed effect repeat measure analysis with a clustered patient-level residual error with unstructured covariance over visits, fixed effects for visit, and all other covariates assumed to have a constant fixed effect over time.

It is anticipated that future data collection will also take place following completion of treatment to assess disease burden and the longer-term impact

For peer review only - http://bmjopen.bmj.com/site/about/guidelines.xhtml

of "Top-Down" vs. "Accelerated Step-Up" treatment approaches on subsequent disease course for these patients.

Conclusions

Currently there is a clear unmet need in the management of IBD, in that treatment strategies – whatever they may be – are typically applied in a one-size-fits-all manner or using "prognostic" markers that have not been shown to be able to guide therapy.

The PROFILE trial is the first biomarker-stratified trial in IBD and will investigate whether a blood-based biomarker, assessed at diagnosis, can stratify patients with CD to receive therapy that is appropriately matched to their subsequent disease course.

If stratification by IBD^{hi}/IBD^{lo} status is demonstrated to improve clinical outcomes by appropriately identifying those patients who require "Top-Down" therapy and those who can be safely managed with "Accelerated Step-Up" therapy, this would represent a step-change in the management of CD and would help make personalised medicine a reality for patients.

Ethics and dissemination

The trial protocol was approved by the East of England - Cambridge South Research Ethics Committee (Ref: 17/EE/03/82). Recruitment for the PROFILE trial began in December 2017 and is currently ongoing at sites around the United Kingdom. On completion of the trial, the data will be analysed and tabulated and a final trial report prepared. Following trial completion and analysis, the results will be presented at scientific meetings and submitted for publication in a peer-reviewed journal. Press releases will be prepared to accompany publication of this trial in order to share the results more widely with the global medical community, trial participants and patient support groups. Reasonable applications for individual clinical trial participant-level data will be considered by the trial team and shared on a controlled access basis if approved. Authorship of final trial outputs will be assigned in

For peer review only - http://bmjopen.bmj.com/site/about/guidelines.xhtml

accordance with guidelines set out by the International Committee of Medical Journal Editors. The SPIRIT reporting guidelines have been used in preparation of this article.[32]

For peer review only

For peer review only - http://bmjopen.bmj.com/site/about/guidelines.xhtml

References

1 Kaplan GG, Ng SC. Understanding and Preventing the Global Increase of Inflammatory Bowel Disease. *Gastroenterology* 2017;**152**:313–321.e2. doi:10.1053/j.gastro.2016.10.020

2 Ng SC, Shi HY, Hamidi N, *et al.* Worldwide incidence and prevalence of inflammatory bowel disease in the 21st century: a systematic review of population-based studies. *Lancet* 2017;**390**:2769–78. doi:10.1016/S0140-6736(17)32448-0

3 D'Haens G, Baert F, van Assche G, *et al.* Early combined immunosuppression or conventional management in patients with newly diagnosed Crohn's disease: an open randomised trial. *Lancet* 2008;**371**:660–7. doi:10.1016/S0140-6736(08)60304-9

4 Schreiber S, Reinisch W, Colombel JF, *et al.* Subgroup analysis of the placebo-controlled CHARM trial: Increased remission rates through 3years for adalimumab-treated patients with early Crohn's disease. *J Crohn's Colitis* 2013;**7**:213–21. doi:10.1016/j.crohns.2012.05.015

5 Hanauer SB, Feagan BG, Lichtenstein GR, *et al.* Maintenance infliximab for Crohn's disease: The ACCENT I randomised trial. *Lancet* 2002;**359**:1541–9. doi:10.1016/S0140-6736(02)08512-4

6 Colombel JF, Sandborn WJ, Reinisch W, *et al.* Infliximab, azathioprine, or combination therapy for Crohn's disease. *N Engl J Med* 2010;**362**:1383–95. doi:10.1056/NEJMoa0904492

7 Khanna R, Bressler B, Levesque BG, *et al.* Early combined immunosuppression for the management of Crohn's disease (REACT): A cluster randomised controlled trial. *Lancet* 2015;**386**:1825–34. doi:10.1016/S0140-6736(15)00068-9

8 Sans M, Román AL, Esteve M, *et al.* Early Use of Azathioprine Has a Steroid Sparing Effect on Recently Diagnosed Crohn's Disease Patients. *Gastroenterology* 2011;**140**:S-109. doi:10.1016/S0016-5085(11)60441-1

9 Cosnes J, Bourrier A, Laharie D, *et al.* Early administration of azathioprine vs conventional management of Crohn's Disease: a randomized controlled trial. *Gastroenterology* 2013;**145**:758–65.

For peer review only

For peer review only - http://bmjopen.bmj.com/site/about/guidelines.xhtml

doi:10.1053/j.gastro.2013.04.048
10 Hart AL, Lomer M, Verjee A, *et al.* What are the top 10 research questions in the treatment of inflammatory bowel disease? *J Crohn's Colitis* 2017;**11**:204–11. doi:10.1093/ecco-jcc/jjw144
11 Lee JC, Lyons PA, McKinney EF, *et al.* Gene expression profiling of CD8+ T cells predicts prognosis in patients with Crohn disease and ulcerative colitis. *J Clin Invest* 2011;**121**:4170–9. doi:10.1172/JCI59255DS1
12 McKinney EF, Lyons PA, Carr EJ, *et al.* A CD8+T cell transcription signature predicts prognosis in autoimmune disease. *Nat Med* 2010;**16**:586–91. doi:10.1038/nm.2130
13 McKinney EF, Lee JC, Jayne DRW, *et al.* T-cell exhaustion, co-stimulation and clinical outcome in autoimmunity and infection. *Nature* 2015;**523**:612–6. doi:10.1038/nature14468
14 Lee J, Biasci D, Noor N, *et al.* PROFILE trial: predicting outcomes for Crohn's disease using a molecular biomarker. *Gut* 2017;**66**:A22–3. doi:10.1136/gutjnl-2017-314472.44
15 Freidlin B, McShane LM, Korn EL. Randomized clinical trials with biomarkers: Design issues. J. Natl. Cancer Inst. 2010;**102**:152–60. doi:10.1093/jnci/djp477
16 Potti A, Mukherjee S, Petersen R, *et al.* A Genomic Strategy to Refine Prognosis in Early-Stage Non–Small-Cell Lung Cancer. *N Engl J Med* 2006;**355**:570–80. doi:10.1056/NEJMoa060467
17 Gomollón F, Dignass A, Annese V, *et al.* 3rd European evidence-based consensus on the diagnosis and management of Crohn's disease 2016: Part 1: Diagnosis and medical management. *J Crohn's Colitis* 2017;**11**:3–25. doi:10.1093/ecco-jcc/jjw168
18 Su C, Lewis JD, Goldberg B, *et al.* A Meta-Analysis of the Placebo Rates of Remission and Response in Clinical Trials of Active Ulcerative Colitis. *Gastroenterology* 2007;**132**:516–26. doi:10.1053/j.gastro.2006.12.037
19 Heap GA, Weedon MN, Bewshea CM, *et al.* HLA-DQA1-HLA-DRB1 variants confer susceptibility to pancreatitis induced by thiopurine immunosuppressants. *Nat Genet* 2014;**46**:1131–4. doi:10.1038/ng.3093

For peer review only - http://bmjopen.bmj.com/site/about/guidelines.xhtml

20 Bretz F, Maurer W, Brannath W, *et al.* A graphical approach to sequentially rejective multiple test procedures. *Stat Med* 2009;**28**:586–604. doi:10.1002/sim.3495

21 Rutgeerts P, Van Assche G, Sandborn WJ, *et al.* Adalimumab induces and maintains mucosal healing in patients with Crohn's disease: Data from the EXTEND trial. *Inflamm Bowel Dis* 2012;**142**:1102–1111. doi:10.1053/j.gastro.2012.01.035

22 Baert F, Moortgat L, Van Assche G, *et al.* Mucosal Healing Predicts Sustained Clinical Remission in Patients With Early-Stage Crohn's Disease. *Gastroenterology* 2010;**138**:463–8. doi:10.1053/j.gastro.2009.09.056

23 Feagan BG, Sandborn WJ, D'Haens G, *et al.* The role of centralized reading of endoscopy in a randomized controlled trial of mesalamine for ulcerative colitis. *Gastroenterology* 2013;**145**. doi:10.1053/j.gastro.2013.03.025

24 Feagan B, Sandborn WJ, Rutgeerts P, *et al.* Performance of Crohn's disease Clinical Trial Endpoints based upon Different Cutoffs for Patient Reported Outcomes or Endoscopic Activity: Analysis of EXTEND Data. *Inflamm Bowel Dis* 2018;**00**:1–11. doi:10.1093/ibd/izx082

25 Daperno M, D'Haens G, Van Assche G, *et al.* Development and validation of a new, simplified endoscopic activity score for Crohn's disease: The SES-CD. *Gastrointest Endosc* 2004;**60**:505–12. doi:10.1016/S0016-5107(04)01878-4

26 Khanna R, Zou G, D'Haens G, *et al.* Reliability among central readers in the evaluation of endoscopic findings from patients with Crohn's disease. *Gut* 2016;**65**:1119–25. doi:10.1136/gutjnl-2014-308973

27 Reinisch W, Colombel JF, D'Haens G, *et al.* Characterisation of mucosal healing with adalimumab treatment in patients with moderately to severely active Crohn's disease: Results from the EXTEND trial. *J Crohn's Colitis* 2017;**11**:425–34. doi:10.1093/ecco-jcc/jjw178

28 Rimola J, Rodriguez S, García-Bosch O, *et al.* Magnetic resonance for assessment of disease activity and severity in ileocolonic Crohn's disease. *Gut* 2009;**58**:1113–20. doi:10.1136/gut.2008.167957

29 Rimola J, Ordás I, Rodriguez S, *et al.* Magnetic resonance imaging for

For peer review only

For peer review only - http://bmjopen.bmj.com/site/about/guidelines.xhtml

evaluation of Crohn's disease: Validation of parameters of severity and quantitative index of activity. *Inflamm Bowel Dis* 2011;**17**:1759–68. doi:10.1002/ibd.21551

30 Ordás I, Rimola J, Rodríguez S, *et al.* Accuracy of magnetic resonance enterography in assessing response to therapy and mucosal healing in patients with Crohn's disease. *Gastroenterology* 2014;**146**. doi:10.1053/j.gastro.2013.10.055

31 Jairath V, Ordas I, Zou G, *et al.* Reliability of Measuring Ileo-Colonic Disease Activity in Crohn's Disease by Magnetic Resonance Enterography. *Inflamm Bowel Dis* 2018;**24**:440–9. doi:10.1093/ibd/izx040

32 Chan AW, Tetzlaff JM, Altman DG, *et al.* SPIRIT 2013 statement: Defining standard protocol items for clinical trials. Ann. Intern. Med. 2013;**158**:200–7. doi:10.7326/0003-4819-158-3-201302050-00583

For peer review only

For peer review only - http://bmjopen.bmj.com/site/about/guidelines.xhtml

Author statement: MP, NMN, FD, KGCS and JCL wrote the full trial protocol. HL, PK, and APS reviewed and edited draft versions of the protocol. The Statistics and Data Analysis sections of the trial protocol were written by SB. LW contributed to the Trial Treatment section of the protocol, and SU contributed to the Radiology section. PAL, EFM, MP, KGCS and JCL were the initiators of this trial. All authors took part in reading and final approval of the manuscript.

Acknowledgements: We are grateful for input and assistance from Carrie Bayliss (Cambridge Clinical Trials Unit) and Dr Amanda Wooding (Cambridge Enterprise) in securing funding for this trial, and to Dr Adrian Mander, Prof Robert Gray, Prof Geert D'Haens, Prof Severine Vermeire, Dr Sharon O'Byrne, Prof Subrata Ghosh, Dr Nick Carroll and Dr Ed Godfrey for helpful discussion regarding the protocol. We are grateful to the patients who provided feedback on the study design and documentation. The protocol has been peer reviewed by the British Society of Gastroenterology IBD Clinical Research Group (BSG IBD CRG). Details of participating centres can be found at http://www.crohnsprofiletrial.com/index.php/investigators/. KGCS is a Wellcome Investigator and an NIHR Senior Investigator. EFM and JCL are supported by Wellcome Trust Intermediate Clinical Fellowships (104064/Z/14/Z and 105920/Z/14/Z respectively).

Patient records: Data are collected via a paper CRF, provided by the trial co-ordination team, and after being input electronically, will be stored in a secured database. Participants will only be identifiable by a trial-specific number in the database. Essential documents will be retained until at least 15 years after the publication of the clinical trial report.

Trial committees: The unblinded data will be presented to the Data Monitoring Committee, who will meet on a regular basis throughout the trial and who are independent from the sponsor. The Data Monitoring Committee will then prepare a report for the Trial Steering Committee who will provide overall supervision of the trial.

For peer review only - http://bmjopen.bmj.com/site/about/guidelines.xhtml

Funding: This trial is funded by the Wellcome Trust via an investment in PredictImmune (200448/Z/16/Z). The Apex Healthcare Consulting survey and biomarker development work described herein was funded by a Wellcome Trust Interim Translational Award (099450/Z/12/Z). The trial is co-sponsored by Cambridge University Hospitals NHS Foundation Trust and University of Cambridge. The sponsors are not involved in study design; collection, management, analysis, and interpretation of data; writing of the report; and the decision to submit the report for publication.

Competing interests statement: PAL, EFM, KGCS and JCL are co-inventors on a patent covering the method of assessing prognosis in IBD. PAL, EFM, and KGCS are co-founders and consultants for PredictImmune. PK and APS are employees of PredictImmune. JCL is a consultant for PredictImmune.

Word count: 3281/4000

For peer review only

Figure legends

Figure 1 – Trial Design

Following biomarker stratification, patients will be randomised in a 1:1 fashion to either “Top-Down” or “Accelerated Step-Up” treatment arms.

Figure 2 – Trial visits for participants.

Patients randomised to “Accelerated Step-Up” will have a total of 5 further trial visits after their initial screening visit. Participants randomised to the “Top-Down” group will be started on Infliximab at week 2. All further Infliximab infusion visits should be aligned to scheduled trial visits wherever possible in order to minimise visit burden for participants. Participants in the “Top-Down” group will also have 5 trial visits and will also attend hospital an additional 4 times for infliximab infusions. Randomisation occurs at week 0.

For peer review only

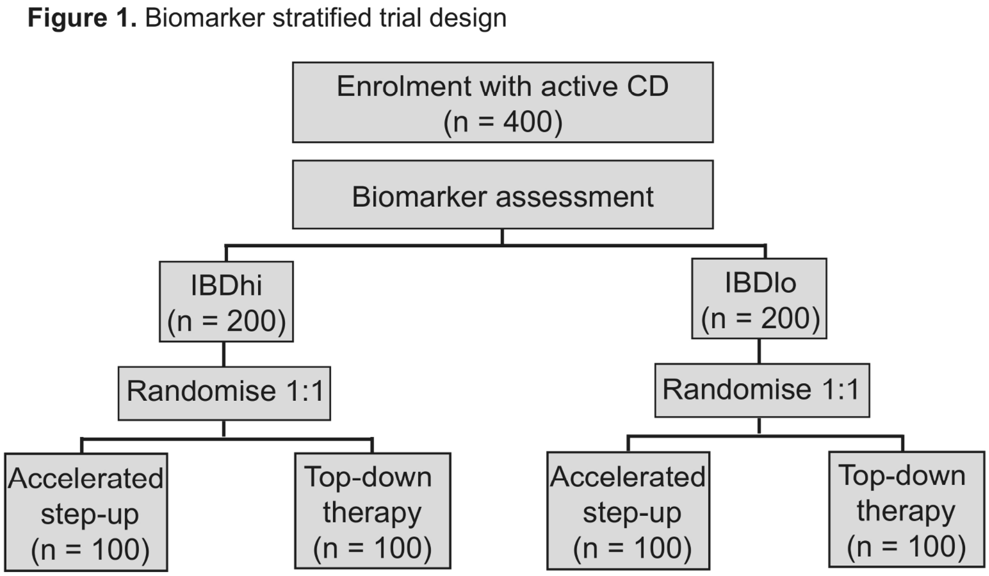

Figure 1 – Trial Design
Following biomarker stratification, patients will be randomised in a 1:1 fashion to either "Top-Down" or "Accelerated Step-Up" treatment arms.

213x124mm (300 x 300 DPI)

For peer review only - http://bmjopen.bmj.com/site/about/guidelines.xhtml

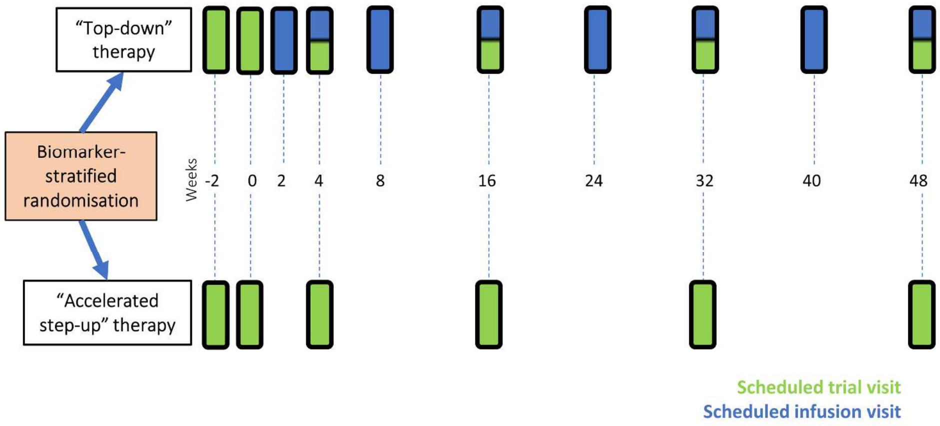

Figure 2 – Trial visits for participants.
Patients randomised to "Accelerated Step-Up" will have a total of 5 further trial visits after their initial screening visit. Participants randomised to the "Top-Down" group will be started on Infliximab at week 2. All further Infliximab infusion visits should be aligned to scheduled trial visits wherever possible in order to minimise visit burden for participants. Participants in the "Top-Down" group will also have 5 trial visits and will also attend hospital an additional 4 times for infliximab infusions. Randomisation occurs at week 0.

125x89mm (600 x 600 DPI)

Supplementary Material

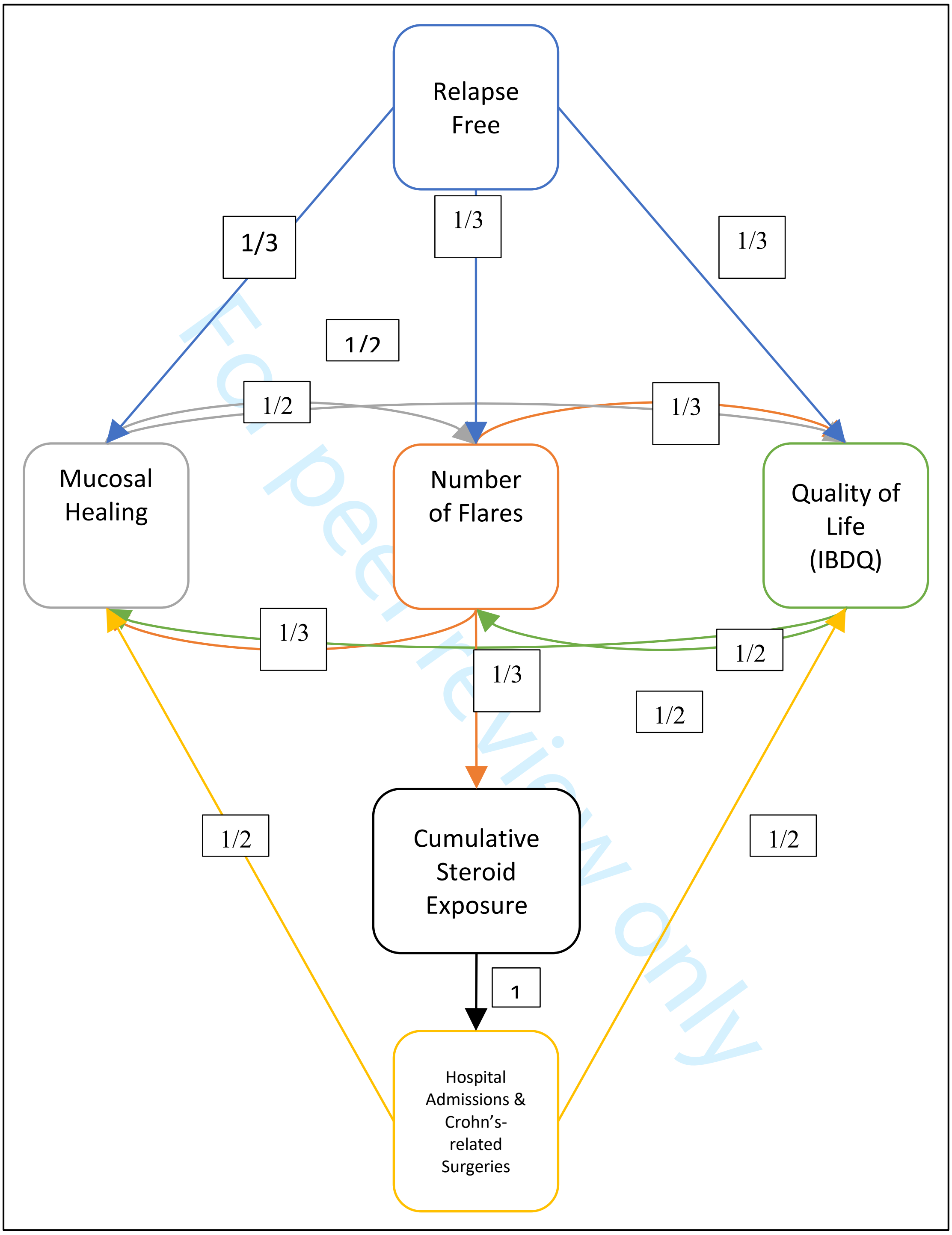

Supplementary Figure 1 – Statistical approach for the PROFILE trial.

The methodology will combine together gate-keeping and Holm-Bonferroni methods in formal hypothesis testing, with the above diagram defining how the significance levels will be transitioned assuming an initial configuration of 5% at the primary endpoint (relapse-free remission) and 0% on all other tests. All the secondary endpoints are continuous variables and will be analysed using a linear regression framework adjusting for baseline covariates.

Reporting checklist for protocol of a clinical trial.

Based on the SPIRIT guidelines.

Instructions to authors

Complete this checklist by entering the page numbers from your manuscript where readers will find each of the items listed below.

Your article may not currently address all the items on the checklist. Please modify your text to include the missing information. If you are certain that an item does not apply, please write "n/a" and provide a short explanation.

Upload your completed checklist as an extra file when you submit to a journal.

In your methods section, say that you used the SPIRIT reporting guidelines, and cite them as:

Chan A-W, Tetzlaff JM, Altman DG, Laupacis A, Gøtzsche PC, Krleža-Jerić K, Hróbjartsson A, Mann H, Dickersin K, Berlin J, Doré C, Parulekar W, Summerskill W, Groves T, Schulz K, Sox H, Rockhold FW, Rennie D, Moher D. SPIRIT 2013 Statement: Defining standard protocol items for clinical trials. Ann Intern Med. 2013;158(3):200-207

		Reporting Item	Page Number
Title	#1	Descriptive title identifying the study design, population, interventions, and, if applicable, trial acronym	1
Trial registration	#2a	Trial identifier and registry name. If not yet registered, name of intended registry	3
Trial registration: data set	#2b	All items from the World Health Organization Trial Registration Data Set	1 (contacts, title), 2 (registration details, countries of recruitment, disease, intervention, study type, sample size, primary and secondary outcomes), 11 (inclusion / exclusion criteria), 17 (ethics review), 24 (sponsor)
Protocol version	#3	Date and version identifier	7

For peer review only

Funding	#4	Sources and types of financial, material, and other support	22-23
Roles and responsibilities: contributorship	#5a	Names, affiliations, and roles of protocol contributors	22
Roles and responsibilities: sponsor contact information	#5b	Name and contact information for the trial sponsor	23
Roles and responsibilities: sponsor and funder	#5c	Role of study sponsor and funders, if any, in study design; collection, management, analysis, and interpretation of data; writing of the report; and the decision to submit the report for publication, including whether they will have ultimate authority over any of these activities	23
Roles and responsibilities: committees	#5d	Composition, roles, and responsibilities of the coordinating centre, steering committee, endpoint adjudication committee, data management team, and other individuals or groups overseeing the trial, if applicable (see Item 21a for data monitoring committee)	22
Background and rationale	#6a	Description of research question and justification for undertaking the trial, including summary of relevant studies (published and unpublished) examining benefits and harms for each intervention	5-7
Background and rationale: choice of comparators	#6b	Explanation for choice of comparators	5-7
Objectives	#7	Specific objectives or hypotheses	9
Trial design	#8	Description of trial design including type of trial (eg, parallel group, crossover,	9

For peer review only

		factorial, single group), allocation ratio, and framework (eg, superiority, equivalence, non-inferiority, exploratory)	
Study setting	#9	Description of study settings (eg, community clinic, academic hospital) and list of countries where data will be collected. Reference to where list of study sites can be obtained	9
Eligibility criteria	#10	Inclusion and exclusion criteria for participants. If applicable, eligibility criteria for study centres and individuals who will perform the interventions (eg, surgeons, psychotherapists)	11
Interventions: description	#11a	Interventions for each group with sufficient detail to allow replication, including how and when they will be administered	13-14
Interventions: modifications	#11b	Criteria for discontinuing or modifying allocated interventions for a given trial participant (eg, drug dose change in response to harms, participant request, or improving / worsening disease)	13-14
Interventions: adherance	#11c	Strategies to improve adherence to intervention protocols, and any procedures for monitoring adherence (eg, drug tablet return; laboratory tests)	14
Interventions: concomitant care	#11d	Relevant concomitant care and interventions that are permitted or prohibited during the trial	13
Outcomes	#12	Primary, secondary, and other outcomes, including the specific measurement variable (eg, systolic blood pressure), analysis metric (eg, change from baseline, final value, time to event), method of aggregation (eg, median, proportion), and time point for each	12

For peer review only

		outcome. Explanation of the clinical relevance of chosen efficacy and harm outcomes is strongly recommended	
Participant timeline	#13	Time schedule of enrolment, interventions (including any run-ins and washouts), assessments, and visits for participants. A schematic diagram is highly recommended (see Figure)	Fig.2
Sample size	#14	Estimated number of participants needed to achieve study objectives and how it was determined, including clinical and statistical assumptions supporting any sample size calculations	9,15
Recruitment	#15	Strategies for achieving adequate participant enrolment to reach target sample size	9
Allocation: sequence generation	#16a	Method of generating the allocation sequence (eg, computer-generated random numbers), and list of any factors for stratification. To reduce predictability of a random sequence, details of any planned restriction (eg, blocking) should be provided in a separate document that is unavailable to those who enrol participants or assign interventions	12-13
Allocation concealment mechanism	#16b	Mechanism of implementing the allocation sequence (eg, central telephone; sequentially numbered, opaque, sealed envelopes), describing any steps to conceal the sequence until interventions are assigned	13
Allocation: implementation	#16c	Who will generate the allocation sequence, who will enrol participants, and who will assign participants to interventions	9,13
Blinding (masking)	#17a	Who will be blinded after assignment to	13

For peer review only

		interventions (eg, trial participants, care providers, outcome assessors, data analysts), and how	
Blinding (masking): emergency unblinding	#17b	If blinded, circumstances under which unblinding is permissible, and procedure for revealing a participant's allocated intervention during the trial	n/a – blinding is to biomarker status not to treatment.
Data collection plan	#18a	Plans for assessment and collection of outcome, baseline, and other trial data, including any related processes to promote data quality (eg, duplicate measurements, training of assessors) and a description of study instruments (eg, questionnaires, laboratory tests) along with their reliability and validity, if known. Reference to where data collection forms can be found, if not in the protocol	22
Data collection plan: retention	#18b	Plans to promote participant retention and complete follow-up, including list of any outcome data to be collected for participants who discontinue or deviate from intervention protocols	22
Data management	#19	Plans for data entry, coding, security, and storage, including any related processes to promote data quality (eg, double data entry; range checks for data values). Reference to where details of data management procedures can be found, if not in the protocol	22
Statistics: outcomes	#20a	Statistical methods for analysing primary and secondary outcomes. Reference to where other details of the statistical analysis plan can be found, if not in the protocol	15, supp. Fig 1
Statistics:	#20b	Methods for any additional analyses (eg,	15-16, supp. Fig 1

For peer review only

additional analyses		subgroup and adjusted analyses)	
Statistics: analysis population and missing data	#20c	Definition of analysis population relating to protocol non-adherence (eg, as randomised analysis), and any statistical methods to handle missing data (eg, multiple imputation)	15-16, supp. Fig 1
Data monitoring: formal committee	#21a	Composition of data monitoring committee (DMC); summary of its role and reporting structure; statement of whether it is independent from the sponsor and competing interests; and reference to where further details about its charter can be found, if not in the protocol. Alternatively, an explanation of why a DMC is not needed	22
Data monitoring: interim analysis	#21b	Description of any interim analyses and stopping guidelines, including who will have access to these interim results and make the final decision to terminate the trial	n/a – no interim analysis is planned.
Harms	#22	Plans for collecting, assessing, reporting, and managing solicited and spontaneously reported adverse events and other unintended effects of trial interventions or trial conduct	14
Auditing	#23	Frequency and procedures for auditing trial conduct, if any, and whether the process will be independent from investigators and the sponsor	n/a - a monitoring plan is currently in the process of being generated - detailing the frequency and scope of monitoring, including of trial conduct. All participating sites will be subject to routine trial specific on-site monitoring.
Research ethics	#24	Plans for seeking research ethics	10

For peer review only

approval		committee / institutional review board (REC / IRB) approval	
Protocol amendments	#25	Plans for communicating important protocol modifications (eg, changes to eligibility criteria, outcomes, analyses) to relevant parties (eg, investigators, REC / IRBs, trial participants, trial registries, journals, regulators)	7
Consent or assent	#26a	Who will obtain informed consent or assent from potential trial participants or authorised surrogates, and how (see Item 32)	14
Consent or assent: ancillary studies	#26b	Additional consent provisions for collection and use of participant data and biological specimens in ancillary studies, if applicable	n/a – no additional consent forms will be used.
Confidentiality	#27	How personal information about potential and enrolled participants will be collected, shared, and maintained in order to protect confidentiality before, during, and after the trial	12-14
Declaration of interests	#28	Financial and other competing interests for principal investigators for the overall trial and each study site	23
Data access	#29	Statement of who will have access to the final trial dataset, and disclosure of contractual agreements that limit such access for investigators	17
Ancillary and post trial care	#30	Provisions, if any, for ancillary and post-trial care, and for compensation to those who suffer harm from trial participation	14
Dissemination policy: trial results	#31a	Plans for investigators and sponsor to communicate trial results to participants, healthcare professionals, the public, and other relevant groups (eg, via publication,	10

For peer review only

		reporting in results databases, or other data sharing arrangements), including any publication restrictions	
Dissemination policy: authorship	#31b	Authorship eligibility guidelines and any intended use of professional writers	17
Dissemination policy: reproducible research	#31c	Plans, if any, for granting public access to the full protocol, participant-level dataset, and statistical code	7 – website link to the full trial protocol.
Informed consent materials	#32	Model consent form and other related documentation given to participants and authorised surrogates	7 – website link to full participant information sheet and consent form.
Biological specimens	#33	Plans for collection, laboratory evaluation, and storage of biological specimens for genetic or molecular analysis in the current trial and for future use in ancillary studies, if applicable	14

The SPIRIT checklist is distributed under the terms of the Creative Commons Attribution License CC-BY-ND 3.0. This checklist can be completed online using https://www.goodreports.org/, a tool made by the EQUATOR Network in collaboration with Penelope.ai

For peer review only
