# Supplementary material for: PRedicting Outcomes For Crohn’s dIsease using a moLecular biomarkEr (PROFILE): protocol for a multicentre, randomised, biomarker-stratified trial
Source: BMJ Open. 2018 Dec 5;8(12):e026767. doi: 10.1136/bmjopen-2018-026767 (PMC6286485; doi:10.1136/bmjopen-2018-026767)
Supplement: Reviewer comments [file bmjopen-2018-026767.reviewer_comments.pdf]

PEER REVIEW HISTORY

BMJ Open publishes all reviews undertaken for accepted manuscripts. Reviewers are asked to complete a checklist review form (**http://bmjopen.bmj.com/site/about/resources/checklist.pdf**) and are provided with free text boxes to elaborate on their assessment. These free text comments are reproduced below.

ARTICLE DETAILS

**TITLE (PROVISIONAL)**	Predicting outcomes for Crohn's disease using a molecular biomarker (PROFILE): protocol for a multi-centre, randomised, biomarker-stratified trial
**AUTHORS**	Parkes, M; Noor, Nurulamin; Dowling, Francis; Leung, Harvey; Bond, Simon; Whitehead, Lynne; Upponi, Sara; Kinnon, Paul; Sandham, Andrew; Lyons, Paul; McKinney, Eoin; Smith, Kenneth; Lee, James

This article was not externally reviewed at BMJ Open. Protocols that have been independently assessed prior to submission to BMJ Open are usually fast-tracked to publication on the grounds that further substantial changes will not be possible. This independent assessment will usually be external, independent review for both a substantial grant award from a non-commercial or government funder and ethics approval.
